# Developing an intervention to improve the quality of childcare centers in resource-poor urban settings: a mixed methods study in Nairobi, Kenya

**DOI:** 10.3389/fpubh.2023.1195460

**Published:** 2023-07-17

**Authors:** Linda Oloo, Helen Elsey, Mary Abboah-Offei, Martin Kiyeng, Patrick Amboka, Kenneth Okelo, Patricia Kitsao-Wekulo, Elizabeth Kimani-Murage, Nelson Langa't, Margaret Nampijja

**Affiliations:** ^1^African Population and Health Research Centre, Nairobi, Kenya; ^2^Hull and York Medical School and Department of Health Sciences, University of York, York, United Kingdom; ^3^School of Health and Social Care, Edinburgh Napier University, Edinburgh, United Kingdom; ^4^Kidogo Early Years, Nairobi, Kenya

**Keywords:** childcare centers, urban informal settlements, intervention development, community of practice, co-creation

## Abstract

**Background:**

Globally, 350 million under-5s do not have adequate childcare. This may damage their health and development and undermine societal and economic development. Rapid urbanization is changing patterns of work, social structures, and gender norms. Parents, mainly mothers, work long hours for insecure daily wages. To respond to increasing demand, childcare centers have sprung up in informal settlements. However, there is currently little or no support to ensure they provide safe, nurturing care accessible to low-income families. Here, we present the process of co-designing an intervention, delivered by local government community health teams to improve the quality of childcare centers and ultimately the health and development of under-5 children in informal settlements in Kenya.

**Methods:**

This mixed methods study started with a rapid mapping of the location and basic characteristics of all childcare centers in two informal settlements in Nairobi. Qualitative interviews were conducted with parents and grandparents (*n* = 44), childcare providers, and community health teams (*n* = 44). A series of 7 co-design workshops with representatives from government and non-governmental organizations (NGOs), community health teams, and childcare providers were held to design the intervention. Questionnaires to assess the knowledge, attitudes, and practices of community health volunteers (*n* = 22) and childcare center providers (*n* = 66) were conducted.

**Results:**

In total, 129 childcare centers were identified −55 in Korogocho and 77 in Viwandani. School-based providers dominated in Korogocho (73%) while home-based centers were prevalent in Viwandani (53%). All centers reported minimal support from any organization (19% supported) and this was particularly low among home-based (9%) and center-based (14%) providers. Home-based center providers were the least likely to be trained in early childhood development (20%), hence the co-designed intervention focused on supporting these centers. All co-design stakeholders agreed that with further training, community health volunteers were well placed to support these informal centers. Findings showed that given the context of informal settlements, support for strengthening management within the centers in addition to the core domains of WHO's Nurturing Care Framework was required as a key component of the intervention.

**Conclusion:**

Implementing a co-design process embedded within existing community health systems and drawing on the lived experiences of childcare providers and parents in informal settlements facilitated the development of an intervention with the potential for scalability and sustainability. Such interventions are urgently needed as the number of home-based and small center-based informal childcare centers is growing rapidly to meet the demand; yet, they receive little support to improve quality and are largely unregulated. Childcare providers, and government and community health teams were able to co-design an intervention delivered within current public community health structures to support centers in improving nurturing care. Further research on the effectiveness and sustainability of support to private and informal childcare centers in the context of low-income urban neighborhoods is needed.

## Background

In rapidly growing cities in low- and middle-income countries (LMICs), changing working and living conditions leave families struggling to care for their children. The World Bank estimates that 350 million children lack quality childcare globally (1). Evidence from population surveys in 61 LMICs showed that up to 17% of under-5 children are left alone at least 1 day a week in East and Southern Africa and up to 29% in South Asia (2). It is estimated that 200 million under-5 in LMICs are at risk of not reaching their developmental potential, largely due to infections, nutritional deficiencies, and a lack of responsive caregiving (3). This is despite global recognition that healthy development in the early years is a pre-requisite for future health and productivity of individuals and societies (4).

Families in cities in LMICs face an unprecedented childcare challenge as gendered work patterns change and both parents work outside the home for long hours (5). With reduced support from the extended family, low-income parents, particularly mothers, are left with limited options for childcare. Many must either take their children to work or leave them at home unattended (6). This undermines child health, already compounded due to detrimental living conditions in deprived urban neighborhoods (7–10) with children exposed to injuries (11, 12), poor nutrition (13, 14), and poor hygiene (10, 15, 16).

Threats to child health are further compounded by the challenges that urban health systems face in meeting the needs of their expanding populations resulting in significant intra-urban inequity in health outcomes (7). While distance may not be a barrier, the limited provision of free, public primary care clinics, open at times when parents are not working, is a significant barrier to access (17). This leads to limited uptake of child health programs among urban poor households (18–20) as exemplified by the finding that only 58% of children in Kenya's slums are immunized (21).

Given changing work patterns and social structures, there is high demand for center-based childcare within cities. Few studies have quantified the demand for center-based care (22). Those that have, have found high demand, for example, 84% of residents in a deprived neighborhood in Dhaka wished to use center-based childcare, with 3.8 (95% CI: 1.4, 10) times higher odds of demand among slum than non-slum households (23). The burgeoning of poor-quality, unregulated centers is clear to see in poor urban neighborhoods across LMICs (1). In one of Nairobi's informal settlements of around 200,000 residents, a rapid survey identified over 50 informal home-based centers (24). Centers frequently consist of one room with poor sanitation and facilities where a woman watches over children for a set fee. While NGO providers are often able to provide better quality childcare than informal providers, they rarely meet parents' needs for long hours of care and our research in Dhaka has found that the sustainability of such externally funded services is a major limitation (23).

Good quality center-based care offers the potential to support healthy child development whilst also allowing parents, particularly mothers to work for an income and older children to remain in school rather than care for younger siblings. There is limited evidence, particularly from Africa and Asia, of the impacts of center-based care on child health, early childhood development (ECD), and wider benefits for mothers, families, and long-term social and economic benefits. Available evidence points to positive impacts on short- and long-term ECD outcomes (25, 26). More recent primary studies have identified improvements in cognitive and socio-emotional skills and long-term impacts on vocabulary (27). Evidence of health impacts is particularly limited, although Behbehani's et al. (28) review of center-based childcare for under-3s found some low-quality evidence of improved immunity and normal growth, especially for those attending for longer (28). In Bangladesh, community-based childcare centers for under-5s in rural areas reduced the risk of all-cause mortality by 44% (95% CI: 20–61%), drowning by 82% (95% CI: 42–94%), and injuries by 88% (95% CI: 61–96%) (29).

Evidence of the social and economic benefits of center-based childcare is considerable, particularly the impact on women's participation in the labor force in high-income countries (HICs) (30, 31) and in LMICs (32–34). Evidence of the impact of childcare centers on family health and wellbeing is limited. However, some limited impact on women's mental health has been identified in India (35).

**Figure 1:**
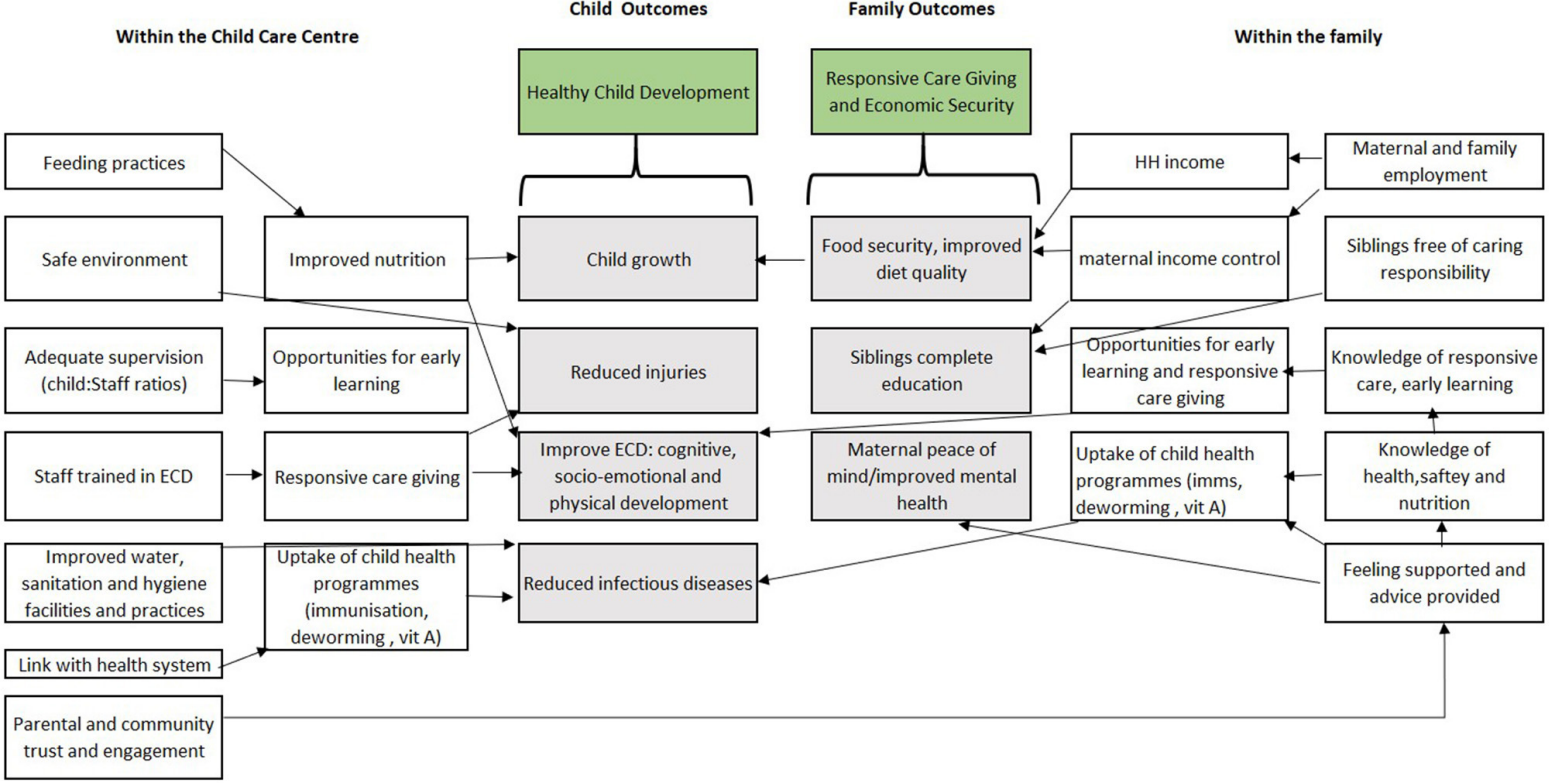
The potential impacts of quality center-based childcare on child and family outcomes.

Importantly, evidence from both HICs (36) and LMICs (37) indicates that low-cost but poor-quality center-based care may worsen ECD outcomes (3) and lead to discontinued breastfeeding and increased infections (28). Figure 1 illustrates the potential benefits of quality center-based childcare to children and their families.

Improving the quality of childcare centers in complex, poor urban neighborhoods is vital if these centers are to benefit, rather than undermine children's healthy development. Given the challenges working parents face in taking up child health programs, childcare centers also offer an ideal setting for outreach to increase access to child health programs such as immunization, deworming, and vitamin A provision among “hard-to-reach” low-income families.

In this study, we present the process of developing an intervention to support center-based childcare in informal settlements in Kenya. Despite the challenges facing public health systems in cities in LMICs, any intervention to support childcare centers must be embedded within this system if it is to be sustainable and with potential for scale-up. The local government-managed community health teams which consist of community health assistants (CHAs) and community health volunteers (CHVs) are a considerable asset within urban areas in Kenya. Their valuable contribution has been recently recognized through the passing of the Nairobi City County Community Health Services Act (June 2021) which provides a monthly stipend and health insurance to CHVs in Nairobi. In recognition of the existing knowledge, experience, and emic perspective of the CHVs and childcare providers, we draw on the Communities of Practice (CoP) model, which, based on the situated learning theory (38), allows peers to share their experiences and practice-based knowledge to learn and improve their practice, rather than through formal instruction or training. The model has been successfully used to harmonize and improve ECD services among various stakeholders in South Africa (39).

The intervention development process is part of a larger study to test the feasibility of the resulting intervention (40). Here, we address the following objectives:

1) To map and assess the childcare environment and provider skills in informal settlements.
2) To co-design with childcare providers, parents, government, and ECD experts a supportive assessment and skill-building CoP approach which can be delivered at scale within informal settlements in Kenya.

## Methods

### Setting

The study was conducted in Korogocho and Viwandani, two informal settlements in Nairobi, Kenya. These two settlements were selected due to their differing characteristics, with Viwandani close to the industrial area being relatively less poor than Korogocho. Viwandani has seven villages, most structures have roofs made from iron sheets and walls from tin and iron sheets. The Ngong River flows to the south of the settlement and is heavily polluted by the industries which are mainly to the north of the settlement. There are also seven villages in Korogocho, and most homes are built of mud and timber and recycled tin cans or sheets as roofing materials. Korogocho is one of the most congested slums in Nairobi with over 250 dwelling units per hectare (41). Both informal settlements have a high proportion of under-5 children and women working outside the home, often in informal employment. Located about 7 km from each other, Korogocho and Viwandani are densely populated with 63,318 and 52,583 inhabitants per square km, respectively. These settlements are characterized by poor housing, poor sanitation, lack of basic infrastructure, insecurity, high crime rate, and poor access to maternal and child health services and healthcare in general (42). Variations in these two communities will support the transferability of study findings to wider urban-poor settings in the East African region.

### Study design

To co-design the intervention, we used a sequential mixed-methods design (43) in combination with the Experience Based Co-Design (EBCD) process (44, 45). EBCD uses a series of interviews, discussions, and observations to share perspectives and issues between different stakeholder groups to develop health service innovations and improvements. EBCD often relies on the use of videos to share the perspectives of different stakeholders to inform intervention design. We mapped childcare centers in two informal settlements to identify their locations for the implementation and feasibility testing phase of the project and to understand their key characteristics to inform the design of the intervention. This was followed by qualitative interviews and group discussions with different stakeholder groups (parents, childcare providers, community health teams, local government officers, and ECD/child health experts), using video to capture key issues that could then be shared during workshops to agree on the design of the intervention.

The data collection period (August 2020 to April 2021) coincided with the COVID-19 pandemic, with subsequent lockdowns and social distancing regulations in Nairobi. During this period, many childcare centers closed in line with COVID-19 guidance for schools. Several changes to our protocol (40) were required. Mapping and assessment of childcare centers, planned as the first activity, had to be delayed until most centers reopened in February 2021. Social distancing requirements meant that we could not hold focus groups using participatory methods with parents, center providers, CHVs, and CHAs and videos to summarize key issues from different participant groups as recommended within the EBCD process (45). Despite waiting for childcare centers to reopen, many remained closed even after restrictions were lifted in both informal settlements selected for the study.

**Figure 2:**
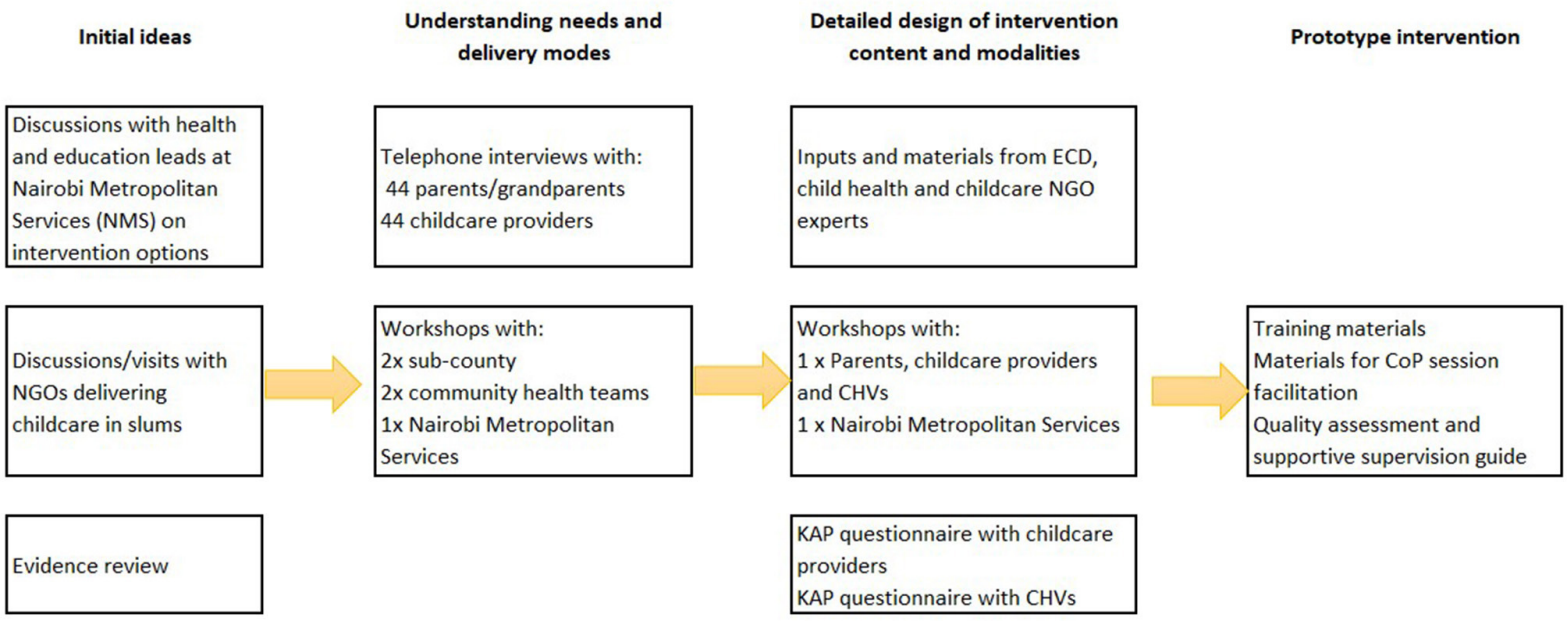
The co-design process.

Figure 2 illustrates the revised co-design process. The initial discussions with sub-county and county officials and non-governmental organizations (NGOs) identified CHVs as an appropriate cadre to deliver the CoP sessions and supportive supervision in informal settlements. Subsequently, qualitative interviews were held by phone (due to COVID restrictions) with parents and guardians using childcare, and with childcare providers. Five workshops were held with parents, community health teams, i.e., CHVs and CHAs, and their sub-County managers and Nairobi City County policy makers. Through a review of published literature, Kenyan ECD and child health policies (46), and discussions held with ECD, child health, and nutrition experts, we identified appropriate content for the intervention training and materials. The NGO partners in the study, Kidogo, who work to support and improve quality among informal childcare centers in Nairobi's informal settlements, shared key materials and childcare center assessment tools, which were adapted and included (see Supplementary Table 8). Following preliminary analysis of qualitative data and reflections on the co-design workshops 1–5, two final workshops (6 and 7) brought together parents, center care providers, government officials, and county and sub-county ECD experts to finalize the CoP model with supportive assessment tools. In the protocol, we planned to map and record brief details of all childcare centers in the two areas and conduct questionnaires to assess the knowledge, attitudes, and practices of childcare providers and CHVs early in the co-design process. However, given the childcare center closures and workload of CHVs due to COVID, the mapping exercise and questionnaires were conducted immediately before the training of CHVs and the start of the intervention. With further training sessions being held over the 4 months of implementation (see Figure 2), we were able to use the questionnaire findings to inform training content and intervention design for the later modules of training.

## Qualitative methods

### Interviews with parents and guardians

With the aid of CHVs in the two informal settlements, we were able to identify childcare providers from different types of centers (see Table 1) using purposive sampling (47). We asked providers from a range of childcare centers to help us identify parents and guardians of the under-5 children using the centers. We then purposively selected mothers, fathers, and guardians of a range of ages, number of children in the household, and different occupations. This allowed us to explore a range of different perspectives and childcare needs during the telephone interviews. Interviews explored participants' current childcare center use, experiences, and preferences, including expected standards and willingness to pay for different services (e.g., provision of hot meals). Details of qualitative interview participants are shown in Table 2 below.

**Table 1:** Typology of center-based childcare providers.

Home-based	Home-based are centers that are within the dwelling units of the childcare center providers. In some cases, the providers hired separate rooms within the plot where they lived and designated the rooms for use as childcare centers. In most cases, however, the same room where the childcare center provider lived also served as the childcare center. Most of the center providers in this category started the childcare center business out of necessity (due to lack of employment) and initially started by looking after neighbors' children and then turned this into business. As a result, the majority do not have any training on ECD or any other childcare-related training.
School-based	School-based are centers that are based within the school system, usually attached to a primary school. In these cases, the schools have a childcare center unit, which also serves as a pre-primary school unit. Most of the care providers in this category are trained ECD and primary school teachers; some are also pursuing degrees in education.
Center-based	Center-based are autonomous centers operating in buildings purposely built for childcare center services. It is not part of a residence building and it does not have a primary school section. Some of the providers in this category are ECD trained, and the rest are not. These are often run by NGOs or as private businesses.
Faith-based	Faith-based are childcare centers that are nested within a church or a mosque and were started by the church or mosque. The teachers are also employed by the respective faith group. Most of the care providers are trained and are employed by the faith organization in which the childcare center is based.

**Table 2:** Qualitative interviews participant characteristics.

**Parent interviews**												
**Attributes**	**Location**		**Occupation**						**Marital status**			
	**Viwandani**	**Korogocho**	**Casual/ daily wage**	**Domestic/ hotel work**	**Office/ salary work**	**Small business**	**No work outside home**	**Prefer not to say**	**Single**	**Separated/ widowed**	**Married**	**Prefer not to say**
Father	2	1	0	0	0	3	0	0	0	0	3	0
Grand-father	1	0	0	0	0	0	0	1	0	0	1	0
Grand-mother	4	6	0	1	0	8	0	1	2	2	4	2
Mother	13	17	8	7	1	12	2	0	11	2	17	0
**Total**	**20**	**24**	**8**	**8**	**1**	**23**	**2**	**2**	**13**	**4**	**25**	**2**
**Childcare provider interviews**									
**Attributes:**	**Location**		**Gender**		**Years of operation**				
**Center type**	**Korogocho**	**Viwandani**	**Male**	**Female**	**0 to**<**3 years**	**3 to**<**5 years**	**5 to**<**10 years**	**10 years plus**	**Total**
Home-based (1 or 2 rooms)	14	16	3	27	6	4	11	9	30
Small center-based, i.e., 3 or more rooms/multiple locations (<24 children)	7	5	1	11	0	4	2	6	12
Large center-based (≥25 children)	2	0	1	1	0	0	0	2	2
	23	21	5	39	6	8	13	17	44

### Interviews with childcare providers

Once all childcare providers were identified by the CHVs, we purposively sampled providers from childcare centers with different characteristics (see Table 1). The interviews with providers explored their experiences of running a childcare center, details of any training and support they had received, how they felt they could improve their centers and the quality of care provided, their interactions with CHVs, and suggestions for the intervention.

Interview guides for both the providers and parents/guardians were translated into Kiswahili and the interviews were conducted by a team of 6 qualitative researchers all fluent in Kiswahili or other local languages to enable good communication with participants. Interviewers received a 5-day training on the study, qualitative interviewing, and ethical issues. The telephone interviews were arranged at a time convenient to the participant and all costs of phone data were covered. Interviews were recorded and transcribed into English soon after the interview. All transcripts were checked by the interviewing team for accuracy. We continued interviewing until similar issues were emerging repeatedly from each participant group, i.e., parents/guardians and childcare providers, and we felt data saturation had been reached (47).

As described earlier, data generated from the interviews were discussed in the co-design workshops (see Table 3) to ensure that the development of supportive supervision and CoPs was grounded in the realities of childcare center provision in informal settlements. Throughout the process, the study team checked back with government officials to clarify any issues raised and gain their feedback on emerging issues.

**Table 3:** Co-design workshops.

**Workshop ID**	**Participants**	**Purpose**	**Date held**
Workshop 1: Sub-county local government (virtual workshop)	10 officials: MoH, Education, nutrition, school health, community health, CHAs, strategist	To understand the potential for using CHVs and gain feedback on initial plans for implementation and content	17.08.2020
Workshop 2: Korogocho community health team (virtual workshop)	10 CHVs 2 CHAs	To discuss CHV motivation for, and the possibility of, adding CoP intervention to CHV/CHA workload and feedback on initial plans	20.08.2020
Workshop 3: Nairobi Metropolitan Services (NMS) (virtual workshop)	6 officials: Director of health, partnerships coordinator, head of communicable diseases, deputy partnerships coordinator, nutrition coordinator, strategy coordinator	To discuss CHV role, training, and support required and gain at the county level	21.08.2020
Workshop 4: Viwandani community health team (virtual workshop)	8 CHVs 1 CHA	To discuss CHV role, training and support required at sub-county level and with the CHVs	24.08.2020
Workshop 5: Sub county (virtual workshop )	9 officials: Sub-county MoH, education, nutrition, school health, community health, CHAs, community strategist		24.08.2020
Workshop 6: Parents, providers and community health team (face-to-face workshop)	4 CHVs, 3 parents, 3 center providers	To share the final draft of the co-designed intervention for feedback	27.01.2021
Workshop 7: Nairobi metropolitan services (NMS) and sub-county (face-to-face workshop)	From the NMS—partnerships coordinator, head of communicable diseases, deputy partnerships coordinator, nutrition coordinator From the 2 sub-counties—MoH, Education, nutrition, school health, community health, CHAs, strategist		29.01.2021

## Mapping and rapid assessment of childcare centers

Field interviewers (FIs), many of whom lived in the two areas, were trained to use OpenStreetMap to map all the childcare centers in the two informal settlements. Each FI was paired with a CHV and together they visited childcare centers within the CHV's catchment area. The CHVs helped to locate the childcare center and subsequently introduced the FI to the childcare center provider. The FI then proceeded with the consent process before administering the mapping tool.

Basic information about the centers was collected by asking the provider questions, observing the facilities and practices in the childcare centers, and checking any records available to ascertain: opening hours; staffing levels; number and ages of children; rooms; hygiene facilities; fees; any organizational/NGO support; and the name of local CHV.

## Knowledge, attitude, and practices questionnaires

### Childcare providers

Center providers who agreed to be involved in the intervention feasibility testing component (*n* = 66) were also asked to complete a questionnaire on their childcare knowledge, attitudes, and practices, specifically: (i) child protection, safety, discipline, and abuse; (ii) stimulating environment; (iii) responsive caregiving; (iv) learning through play; (v) health; (vi) nutrition; (vii) water, sanitation, and hygiene; and (iv) business and administration.

### Community Health Volunteers

Before the initial intervention training session, the CHVs allocated by the sub-county to deliver the intervention (*n* = 22) were asked to complete a brief questionnaire to assess their knowledge in relation to (i) child protection, safety, discipline, and abuse; (ii) stimulating environment; (iii) responsive caregiving; (iv) learning through play; (v) health; (vi) nutrition; and (vii) water, sanitation, and hygiene. In addition, they were asked about their current practices of visiting childcare centers in their catchment areas (if at all) and their motivation to facilitate group sessions and provide support to the center providers (see Supplementary material for all questionnaires).

#### Analysis

An initial rapid analysis of parent and childcare provider interview transcripts was conducted using codebook thematic analysis to identify key areas to include in intervention design. The 5 areas of the Nurturing Care Framework were used as predetermined codes and structured codebooks to analyze from a deductive perspective and then further themes were added as they emerged inductively from the data (48) to structure the rapid analysis: (i) Opportunities for early learning; (ii) good health; (iii) adequate nutrition; (iv) responsive caregiving; and (v) safety and security. The process highlighted additional themes on the community context, center infrastructure/environment, and considerations for intervention delivery. The rapid qualitative analysis supported a clear specification of the types of childcare providers (see Table 3), and this informed the data to be collected during the quantitative mapping of childcare centers. The rapidly identified issues and areas were presented and discussed in the final co-design workshops 6 and 7 held in January 2021. A detailed coding framework grouped around these themes was then applied to all transcripts using NVIvo 2020 software. Detailed notes from the co-design workshops were added to the qualitative analysis and coded using the same themes. Attributes were set for all the respondents based on gender and location (informal settlement) and for the parent/guardian interviews: the relationship to the child; marital status; and occupation; and for the provider interviews: the type (see Table 3) and years of operation of the center. The attributes aided the analysis team to explore different patterns in knowledge and practice between different types of centers and for parents and guardians with different characteristics. Data from the mapping exercise and the KAP questionnaires with CHVs and childcare providers were analyzed using descriptive statistics in Stata Version 17.

## Ethics

This study was approved by Amref Health Africa's Ethics and Scientific Review Committee (Ref: P7802020) and the University of York (Ref: HSRGC).

Consent forms and data, including the location of childcare centers, were kept confidential and locked away to ensure that childcare center providers' privacy was protected.

## Results

The mixed methods results of the brief survey of center characteristics, knowledge, attitudes, and practices surveys and qualitative interviews with parents, guardians, and center providers are presented showing the strengths and challenges of the childcare centers in addressing the nurturing care domains within the context of informal settlements. The meta-inference from these findings and the potential solutions identified during the co-design workshops and how these culminated in the final intervention are presented along with the details of the co-designed intervention.

In total, 129 childcare centers were identified, 55 in Korogocho and 77 in Viwandani. All consented to provide essential information about their centers which is presented in Table 4.

**Table 4:** Characteristics of all mapped child-care centers in Korogocho and Viwandani.

**Variable**	**Category/ Summary statistic**	**Home-based**	**Center-based**	**School-based**	**Faith-based**	**Totals**
Location Number of centers	Korogocho *N* (% within Korogocho)	5 (10%)	5 (10%)	38 (73%)	4 (8%)	52 centers
	Viwandani *N* (% within Viwandani)	40 (52%)	9 (12%)	23 (30%)	5 (6%)	77 centers
	**Total** ***N*** **(% in both sites)**	**45 (35%)**	**14 (11%)**	**61 (40%)**	**9 (7%)**	**129 centers**
Children per center	Mean (sd)	8.5 (5.7)	36.9 (27.2)	47.3 (56.6)	29.8 (20.0)	31.4 (43.8)
	Range (min. to max.)	1–25	2–105	6–429	7–68	1–429
	**Total number of children**	**383**	**517**	**2,885**	**268**	**4,053 children**
Children 0–3 y	*N* (% per center type)	304 (79%)	144 (28%)	479 (17%)	49(18%)	976 children
Children >3 yrs	*N* (% per center type)	79 (21%)	373 (72%)	2,406 (83%)	219 (82%)	3,077 children
Sex (boys)	*N* (% per center type)	181 (47%)	252 (49%)	1,460 (51%)	122 (46%)	2,015 (50%)
Caregiver: child ratio	Mean caregiver: child by center type	1:8	1:22	1:30	1:21	1:22
Years of operation	0–2 yrs (% per center type)	24 (53%)	4 (29%)	9 (15%)	2 (22%)	39 (30%)
	3–5 yrs	4 (9%)	5 (36%)	12 (20%)	2 (22%)	23 (18%)
	6–10 yrs	11 (24%)	2 (14%)	12 (20%)	3 (33%)	28 (22%)
	>10 years	6 (13%)	3 (21%)	28 (46%)	2 (22%)	39 (30%)
Charges per day (ksh)	Mean (sd, range)	56.4 (17.3, 30–100)	47.1 (24.9, 10–100)	57.5 (91.4, 0–500)	33.1 (20.6, 10–170)	1 (1–2)
	Median and IQR and full range for each center type	50 (50–70)	45 (30–50)	30 (25–50)	20 (20–50)	
Support from any organization	Answering Yes *N* (% per center type)	4 (9%)	2 (14%)	17 (28%)	2 (22%)	25 (19%)
Provider trained in ECD	Answering Yes *N* (% per center type)	9 (20%)	10 (71%)	53 (87%)	8 (89%)	80 (62%)
Interested in joining group meetings and assessment/supervision	Answering Yes *N* (% per center type)	45 (100%)	14 (100%)	61 (100%)	9 (100%)	129 (100%)
Know the CHV for their catchment area	Answering Yes *N* (% per center type)	44 (98%)	13 (93%)	52 (85%)	9 (100%)	118 (92%)

Of the 129 centers mapped, almost two-thirds were in Viwandani where small home-based centers dominated. These home-based centers received less external support and were less likely to have had any training in ECD than other center types. This confirmed the information shared during the co-design workshops with community health teams that the home-based centers had poor standards and the (predominantly) women running them were untrained in child health and development and unsupported (Workshops 2, 4, and 6). In light of this, a key decision in the intervention development was to exclude the school-based centers and focus instead on the home-based, slightly larger centers and faith-based centers. Of the 68 centers that fell into these categories, 66 agreed to be part of the implementation and feasibility testing of the intervention and completed the more detailed questionnaire (see Table 5).

**Table 5:** Questionnaire results: knowledge, attitudes, and practices of childcare providers.

			**Home-based**	**Center-based**	**Faith-based**	**Total**
			***N*** = **45** ***n*** **(%)**	***N*** = **14** ***n*** **(%)**	***N*** = **7** ***n*** **(%)**	***N*** = **66** ***n*** **(%)**
Center provider age	Years mean (SD)		39.9 (9.4)	39.1 (9.9)	44.3 (14.0)	40.2 (10.0)
Center provider sex	Female *n* (%)		45 (100%)	12 (86%)	6 (86%)	63 (95%)
Provider highest education level	None		3 (7)	0 (0)	0 (0)	3 (5)
	Primary	24 (53)		2 (14)	1 (14)	27 (41)
	Secondary	17 (38)		7 (50)	3 (43)	27 (41)
	Tertiary	1 (2)		5 (36)	3 (43)	9 (14)
Location of center	Korogocho		7 (16)	5 (36)	3 (43)	15 (23)
	Viwandani	38 (84)		9 (64)	4 (57)	51 (77)
Opening time	5:00–6:00 a.m.		3 (7)	0 (0)	0 (0)	3 (5)
	6:00–7:00 a.m.		30 (67)	7 (50)	3 (43)	40 (61)
	7:00–8:00 a.m.		9 (20)	5 (36)	4 (57)	18 (27)
	8:00–9:00 a.m.		3 (7)	2 (14)	0 (0)	5 (8)
Closing time	4:00–5:00 p.m.		1 (2)	4 (29)	3 (43)	8 (12)
	5:00–6:00 p.m.		9 (20)	4 (29)	2 (29)	15 (23)
	6:00–7:00 p.m.		24 (53)	4 (29)	2 (29)	30 (45)
	7:00–8:00 p.m.		7 (16)	2 (14)	0 (0)	9 (14)
	8:00–9:00 p.m.		4 (9)	0 (0)	0 (0)	4 (6)
Weekend operation	Open on Saturdays		38 (84)	7 (50)	1 (14)	46 (70)
	Open on Sundays		10 (22)	0 (0)	0 (0)	10 (15)
Charges per day (KES)	Median (IQR)		50 (50–70)	45 (30–50)	20 (20–50)	50 (30–50)
	Range		30–100	10–100	10–170	10–170
Early learning	Each child has an opportunity to play with a toy or other object		10 (22)	6 (43)	2 (29)	18 (27)
Responsive care	Children must be treated harshly for them to develop well	Always	3 (7)	0 (0)	0 (0)	3 (5)
		Sometimes	17 (38)	6 (43)	1 (14)	24 (36)
		Never	25 (56)	8 (57)	6 (86)	39 (59)
	Children encouraged and supported to eat their food	Every meal	40 (89)	11 (79)	7 (100)	58 (88)
		Leave children to eat on their own	5 (11)	3 (21)	0 (0)	8 (12)
Nutrition	Children receive morning uji (porridge) and lunch	Both uji and lunch	43 (96)	13 (93)	7 (100)	63 (95)
		Lunch only	1 (2)	0 (0)	0 (0)	1 (2)
		No meals	1 (2)	1 (7)	0 (0)	2 (3)
	Confident to advise parents on healthy diet		33 (73)	12 (86)	5 (71)	50 (76)
	Meal planning with diverse foods		5 (11)	6 (43)	1 (14)	12 (18)
Health	Conduct daily health checks		36 (80)	13 (93)	6 (86)	55 (83)
	Know what to do if a child is sick		43 (96)	14 (100)	7 (100)	64 (97)
	Know immunization status of all children		39 (87%)	11 (79%)	5 (71%)	55 (83%)
Safety	Child should be kept in sight		45 (100)	45 (100)	45 (100)	45 (100)
	Provider should check and remove hazards		45 (100)	14 (100)	7 (100)	66 (100)
	In the past 2 weeks, how many times have children been physically punished in the center	Daily	6 (13)	0 (0)	0 (0)	6 (9)
		Once/twice a week	16 (36)	2 (14)	3 (43)	21 (32)
		Never	23 (51)	12 (86)	4 (57)	39 (59)
Management	Prepares a budget at the start of each week		16 (36)	7 (50)	5 (71)	28 (42)
	Provider tracks income/expenses in their center		16 (36)	9 (64)	6 (86)	31 (47)
	Attendance register is kept		16 (36)	13 (93)	7 (100)	36 (55)
	Business license displayed		0 (0)	2 (14)	1 (14)	3 (5)

## Early learning

**Figure 3:**
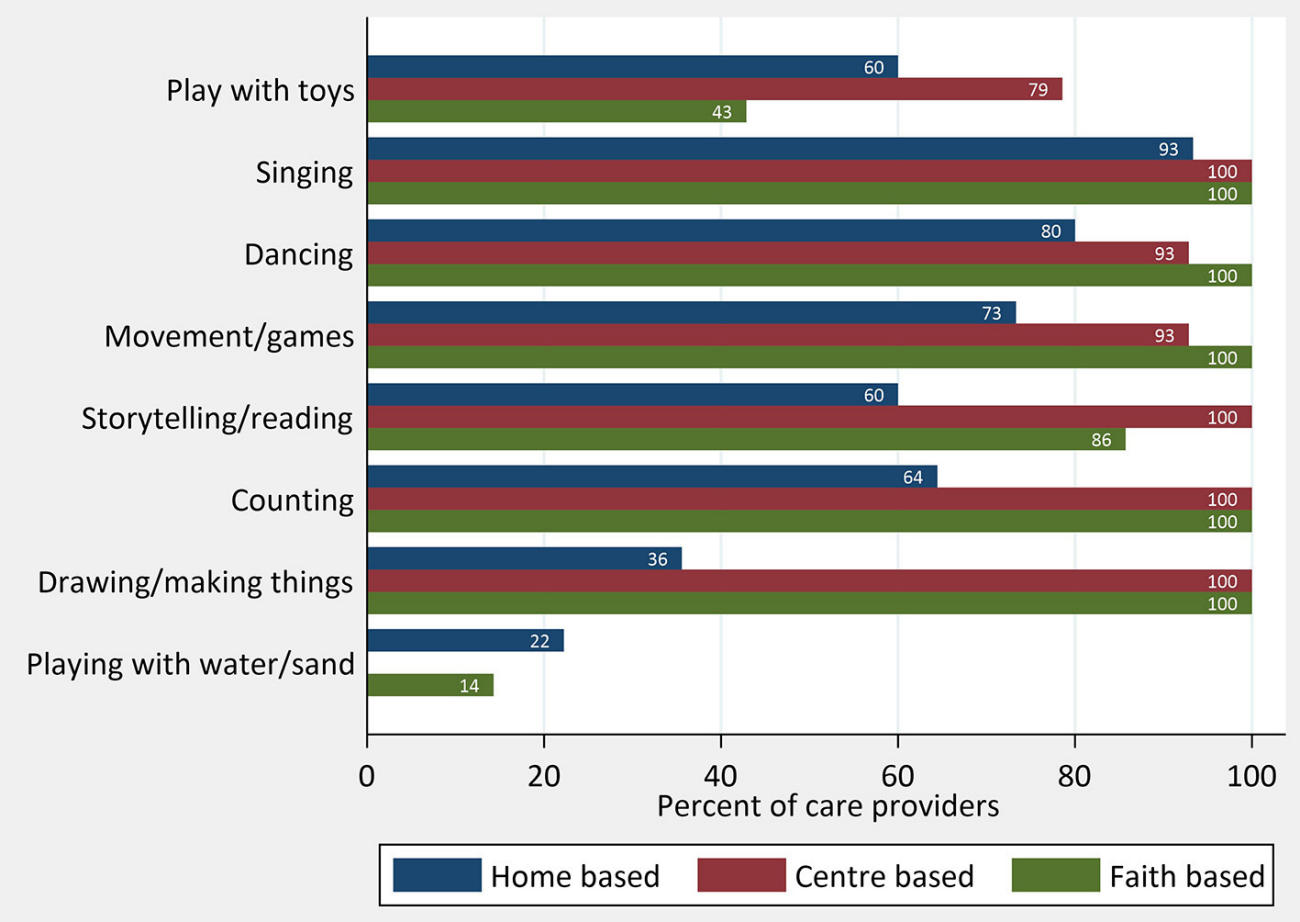
Learning through play: activities in a typical day, by center type.

Both the qualitative interviews and questionnaire results provided insights into early learning practices and perceptions. Most providers talked about how they sang and played with the children using whatever toys and space they had. The questionnaire results reinforce this with singing, dancing, and movement games which are the most common activities reported, particularly in the home-based centers (see Figure 3). Some providers in smaller home-based centers had found ways to create space for play despite the constraints they faced:

“*My house has a space for play because I remove the table. I have two large seats, and a cupboard. I create room in the middle for them to play.”* (Provider K20, home based childcare centre, female, 1–2 years running the centre, Korogocho).

Parents identified multiple gaps in the skills of providers, and even when some providers were skilled in learning through play, they lacked experience in responding to health, nutrition, and hygiene needs of the children.

“*All that happens at that daycare centre… including playing with children… the children know nursery rhymes such as ‘gari ya moshi'. She is very okay with that because she has enough space. The problem with her is not giving the child food on time and not changing the child's diapers.”* (Parent K15, female, married and living with husband, small business owner, Korogocho).

Some providers, particularly those in larger centers, mentioned reading and writing with the older children and described how children had toys and space to allow play:

“*Mostly we group them, there those who like to play and moving around. They play with playing materials like blocks, dolls and toy cars and at certain times they read orally. You write with bold letters you read to them, and they recite so they can get the concept of reading.”* (Provider V6, female larger child-care centre, 3–4 years running the centre, Viwandani).

The lack of play materials was apparent from the questionnaire results with only 22% of home-based and 29% of faith-based providers reporting that children had a toy or other appropriate object to play with. This was slightly higher among the larger centers (43%) (see Table 5). Parents frequently commented on the lack of play and learning materials. Those who were able to, took their own toys for their child to play with at the childcare centers.

Many parents saw the childcare center as an important step in the transition to primary school. They valued the skills of the providers in playing and teaching their children.

“*So she can interact with other children, to learn how to walk, talk and he goes to school it will be easy”* (Parent V2, female, unemployed, married and living with husband, Viwandani).

The lack of training of providers, particularly younger women without children of their own, was identified by many parents as a barrier to responsive caregiving and the ability to support children to learn and develop.

“*More teachers who are trained in childcare should be brought on board, so that the children who are about to join Baby Class can be taught how to be with other children. They can also be introduced to what they will be taught in the next level so that they can know… the day-care service provider is mainly playing the role of a parent right now, but it would be good if she would get more training.”* (Parent K5, female, works outside home as a casual laborer, married and living with husband, Korogocho).

Sub-county and county-level workshop participants focused on the need to create a healthy environment and protect and promote child health, with less of an emphasis on ECD. However, during workshop 5 with sub-county officials, there was a recommendation that the intervention could do much to encourage center providers to make use of any local materials they could find to improvise playing material for child stimulation. The importance of learning through play was identified by participants in workshop 6 who were keen to ensure that providers had these skills to allow their children to develop their skills and talents. Some of the attitudes toward interactions with children were illustrated in the responses to the CHV questionnaire with 27% (6 CHVs) feeling there was no need to show an interest in children's conversation and 32% (7 CHVs) believing very little talk was needed with under-5 children (see Table 6).

**Table 6:** Questionnaire results: knowledge, attitudes, and practices of community health volunteers delivering the intervention.

**Characteristics**	**Sex**	**Education**
*N* = 22 CHVs	Female: 20 (91%)	Primary: 6 (27%) Secondary: 14 (64%) Tertiary: 2 (9%)
**Care domain**	**Question/item**	**Response** ***n*** **(%)**
Early learning	Children learn through play	22 (100%)
Responsive caregiving	Appropriate communication/interaction practices (answering yes)	Very little talk or no conversation needed with children: 7 (32%) Shouting at/speaking angrily is appropriate: 2 (9%) No need to show interest in children's conversations: 6 (27%) Children should be allowed to express themselves freely: 20 (91%) Children's needs must be responded to: 21 (95%)
Nutrition	Children should be fed with a variety of foods rotated on different days:	Always 22 (100%)
	It is important that children are served warm (not cold) food	Agree strongly 21 (95%) Agree a little 1 (5%)
	Caregivers should have knowledge about a balanced diet, and the different food groups	Agree strongly 22 (100%)
Health	It is important to know about the immunization status of children in daycare	Agree strongly 22 (100%)
	Center providers should be knowledgeable on how to do a daily health check and conduct first aid in case of an emergency	Agree completely 21 (95) Agree a little: 1 (5)
	CHVs able to demonstrate correct knowledge of immunization schedule	21 (95%)
Hygiene	CHVs able to demonstrate correct knowledge of handwashing (with soap, after changing diapers, before meals) and sharing utensils during eating	22 (100%)
	Handwashing after playing with soiled toys	17 (77%)
Safety and security	Children must be handled harshly for them to develop well	Never: 18 (82%) Always: 1 (5%) Sometimes: 3 (14%)
	How should children who misbehave be corrected? *(Multiple response)*	Physical punishment: 2 (9%) Verbal punishment: 5 (23%) Distract child: 2 (9%) Explain wrongs calmly: 18 (82%)
CHVs' current interaction with childcare centers	Currently support/advise center providers	21 (95%)
	How often do you childcare providers?	Weekly: 13 (62%) Monthly: 6 (29%) Less than once/month: 2 (10%)
CHV's motivation and skills	Do you feel motivated to support daycare centers within your role as a CHV?	Strongly motivated: 16 (73%) Motivated: 6 (27)
	Do you feel you have the required competencies to support daycare providers?	I am very competent: 3 (14%) I have some of the competencies required: 19 (86%)
Concerns about intervention	Barriers in supporting childcare centers *(Multiple responses)*	Lack of time due to workload: 4 (18%) Lack of training: 12 (55%) Lack of motivation: 4 (18%) Childcare providers not interested: 10 (45%) Lack of tools/resources: 15 (68%) Lack of guidance: 5 (23%)

## Responsive care

Many parents and grandparents emphasized that their main motivation for choosing a childcare center was the caring nature of the provider and most were confident in the abilities of their chosen care provider to provide responsive and caring attention to their children. However, high child-to-caregiver ratios were frequently mentioned, and parents were concerned about how this impacted the responsiveness of the provider to their children's needs. The mapping exercise found the highest child-to-provider ratios of 1:30 among the school-based centers with some centers having over 400 children. Home-based centers had a mean ratio of 1:8. However, there were some outliers with over 20 children and only one provider (see Table 4).

High child-to-provider ratios were raised as key concerns during interviews with parents and the workshops with community health teams (Workshops 2, 4, and 6). Parents identified the negative impacts of these high child-to-provider ratios and expressed concern about how responsive single providers could be, particularly with many children of different ages:

“*That is the biggest challenge like I told you. They need to be like two or three teachers. Because what happens is that the older ones tend to be kept on the side as the young kids are taken care of. She might have two or three children crying and she is alone. She may carry one of them while she the other crying ones continue. The other challenge is on feeding them because they are all in her custody and she is all alone.”* (Parent K5, female, married and living with husband, daily wage earner, Casual laborer outside home, Korogocho).

Being responsive during feeding was an area that was explored in the questionnaire and the majority of providers reported supporting children to eat. However, 11% of providers in home-based and 21% of providers in larger centers reported leaving children to eat on their own. A relatively high proportion of home-based and center-based providers felt children should be treated harshly, with only 56 and 57%, respectively, saying this should never happen, compared to 86% of faith-based centers reporting this never happened in their centers.

## Nutrition

While the questionnaire responses indicated that a high proportion of all providers were able to provide *uji* (porridge) and lunch, within the qualitative data, both parents and providers emphasized their concerns regarding the ability to ensure this level of provision of food at the childcare centers. Within the centers, there were different approaches to the provision of meals depending on the provider's ability to store and heat food. Some parents gave the providers money in addition to the regular fees to buy and prepare cooked food, but the majority in both informal settlements provided pre-packed food for their children. Providers, particularly those from home-based centers, found the pre-packed food difficult to manage with some children having more and better food while others had very little or nothing.

“*I have a big challenge here because there are those who bring food and a parent might come and tell me they don't have food. I make sure they all eat, but some don't come with food and I can't let them starve the whole day.”* (Provider V9, Homebased 1 or 2 rooms, running centre 5–10 years female, Viwandani).

“*The centre is a small room, and the children are around 30 or 40. I am not so sure how many children they are but they are very many. So, they really squeeze in there. They don't have toys there, and sometimes there is no food. They used to be given porridge there but now they are not given*.” (Parent K9, female, married living with husband, daily wage Korogocho).

Our qualitative analysis highlighted the limited nutritional variety in the foods provided by both parents and providers. Rice, *ugali* (Kenyan staple of cooked maize meal), and *uji* (porridge) were mentioned most frequently. Occasionally, providers and parents were able to add cabbages, beans, and spinach depending on available funds. The questionnaire pointed to gaps in provider knowledge on a healthy diet, particularly in advising parents on what they should bring for their children or feed them at home. Few providers were able to plan meals with diverse food for the week ahead (see Table 5).

Given the timing of our study, participants frequently discussed the impacts of COVID lockdowns. Without the ability to earn a daily wage, many families shared that they were unable to feed their children. For some families, lack of food at home meant that any provision of food at a childcare center was reason enough to send them there, despite other factors:

“*I saw other children being given porridge and being taken care of well but some of the teachers were pinching the children, and beating them. But for me, of importance was food. I thought they were feeding them better.”* (Parent K16, female, married and living with husband, small business owner, Korogocho).

Financial challenges of obtaining sufficient cooking fuel, as well as care in cooking food, were identified as constraints to cooking food appropriately and safely. These concerns resulted in parents packing their own food for their children.

“*Most of the childcare centre [providers] simply cook rice and beans for the children. What I don't know is if the beans are usually well-cooked or not. With rice there is no problem but as for beans you never know if they are given cooked beans. So we preferred to pack food for them.”* (Parent K13, female married not living with husband, daily wage earner, Korogocho).

## Health

Given the environment of the informal settlements, many parents did voice concerns about the ability of the centers to maintain hygiene and a clean environment for children to eat, sleep, and play. In particular, they were concerned about changing children's diapers, providing clean and sufficient mattresses, floor coverings and bedding, keeping children clean, ensuring hand washing, and provision of clean drinking water.

“*They should also get a water tank where they can store water for using, like for children to wash their hands.”* (Parent V3, female, married and living with husband, small business owner, Viwandani).

The co-design workshops with CHVs and CHAs reinforced parents' concerns about the centers. Participants commented on how dirty and unhygienic many of the centers were, particularly the home-based centers where the available space was frequently a single room where the center provider lived and also operated the business. Participants shared that: “*You find children sleeping in one corner, there are used potties in another corner and food in another corner”* (Workshop 4: participant).

Parents were particularly concerned about the hygiene standards in storing and preparing food in the centers. They frequently expressed concerns that the childcare provider was not storing the food in a clean area and protecting it from contamination. This was mentioned most frequently in relation to the home-based centers.

“*When I left my child with neighbours, every week my child would fall ill with common colds and other diseases such as coughs. During cold season, he would be left to play with water and then the next week, he is unwell. When I started to take him to a daycare centre I stayed for about two months, for the first time, without having taken him to the hospital.”* (Parent K14, Not married, female, small business owner, Korogocho).

Underlying this theme was the overriding influence of the slum context and the economic hardship facing families. Several parents explained how they were using childcare centers that they felt were a risk to their child's health simply because they were cheap and what they could afford. Frequently, the challenges facing the centers were due to the poor slum environment around the centers.

“*They border a sewer line. Before they fenced the school, children used to fall into the sewer. Then the children have to breathe in the fumes from that sewer line. The fumes make them sick; my children have blocked noses from that”* (Parent K9, female, married and living with husband, daily wage earner Korogocho).

Regarding minor ailments, injuries, and emergency care, parents and providers described the challenges of getting appropriate and timely care. For parents working long hours far away from home and the childcare center, there was great concern that their children would remain untreated until they returned from work when it would be too late to find a healthcare center open. Several parents recommended that childcare centers should keep first aid kits and basic medications.

“*I told you about a pharmacy in the daycare for when a child is not feeling well. When they call you when your child is not feeling well you leave everything to come and take your child to the hospital but if they would have a pharmacy there to give first aid you can come in the evening and see what you will do”* (Parent V3, female, married and living with husband, small business owner, Viwandani).

During the interviews with providers, most focused on the challenges of running their centers rather than the health of the children. However, the questionnaire results indicated that 97% of providers felt they knew what to do if a child was sick and 83% said they checked the children's health each day and knew the immunization status of the children in their care. Several of the more experienced providers from both home-based and larger center-based care mentioned referring parents or even taking the children themselves to local clinics for immunizations or check-ups:

“*You find the child is underweight and the child doesn't have a good diet, there is a hospital called ‘Provide, we advise the parent to take the child within Korogocho and if she is lucky there is these protein sachets that they are given to eat so they regain the weight'.”* (Provider K2, Home-based, running center for 5–10 years Korogocho).

The need for providers to be trained to manage child health, including checking immunization and knowing what to do if a child was sick, was identified as an important part of the support needed by childcare providers by 95% of the 22 CHVs completing the KAP questionnaire.

## Safety and infrastructure

A priority for parents during Workshop 6 and a frequent theme within the qualitative analysis was the safety of their children. The high level of crime and low social capital within the slum environment meant parents were scared to leave their children with neighbors, and few had relatives nearby to provide trusted care.

“*When I go to hustle for a job, leaving her with another person is another source of stress. You are better off leaving her in a place where you know you will not find him in danger. You know neighbours [xxx] have many issues, so it is better to leave the child where you are assured of his safety”* (Parent K13, female, married and not living with husband, daily wage earner, Korogocho).

“*I trust her because when I go to work, I can leave her with my baby and I know my baby will be safe. I have to leave her in a place that I deem safe, she will not stay hungry. Those are what I look out for. A place where she will not be defiled, and she will not stay hungry.”* (Parent K7, female, married and living with husband, daily wage earner, Korogocho).

“*When I am at work, like I said she should be given food, when she falls asleep, she is put to bed, and when I am at work, I don't fear news such as, ‘your child has been defiled'.”* (Parent K6, Not married, daily wage earner, Korogocho).

“*I saw a certain lady who used to take her child there and I wanted a place where my child could spend the whole day, so that I would be secure in knowing that my child was safe in a certain place, where I can go and pick him from when I am back. I didn't even bother about the environment. All I cared about was a place where I could leave my child.”* (Parent K14, female, married but not living with husband, daily wage earner, Korogocho).

These concerns for the safety of the child were expressed by providers, particularly in relation to managing the collection of children. With parents working long hours, agreeing on who should pick children when the parent was delayed was a real concern. Providers who had been running centers for many years explained their policies for pickups. However, managing these issues was particularly challenging for smaller home-based and inexperienced center providers.

“*The parents bring them and when they leave, they come back so late. We agree with her in the evening they can't tell anyone to come and pick the child in the evening, so she has to come with the one who will be picking her child and tell me when they are late this person should take my child”* (Provider K16, Female, home-based, operating for more than 10 years, Korogocho).

The crowding of the childcare centers, particularly the home-based ones, was frequently highlighted by parents as a situation that undermined the safety and healthy development of children.

## Management challenges

The harsh economic realities facing families in informal settlements meant that many providers struggled to run their childcare centers as viable businesses. There were many examples of providers working long hours, accepting under-payment and late payment, and providing food for children when their parents could not do so. The provider questionnaires showed how home-based providers worked particularly long hours with some starting as early as 5 am and closing at 9 pm. Furthermore, 84% of home-based providers opened on Saturdays compared to 50% among larger centers and 14% among faith-based centers, and only home-based centers operated on a Sunday.

Even providers who had been in business for over 5 years explained how they frequently took responsibility for the children when parents struggled to pay, feed, or pick them up on time. The need for providers to play this role was clearly heightened by the economic conditions during the COVID pandemic. However, the unreliability of informal work and lack of economic opportunities were factors identified as influencing the financial viability and management of the centers independent of the pandemic.

“*So, let's say it's a job, the parents go and work overnight and the next day they come at around 11 a.m. Sometimes even if they left their phone number, it is not useful because they change the number so that you can't reach them. So, I have passed through this with like five parents. In fact, I am forced to stay with them [the children] like mine and when they return, they don't give me thanks.”* (Provider V1, female, home-based centre operational for 5–10 years, Viwandani).

Managing centers in these conditions was clearly challenging. For instance, home-based centers had fewer good management practices such as weekly budgeting, tracking income and expenditure, and keeping an attendance register (see Table 5).

## Community health systems and support for childcare centers

The qualitative interviews and mapping exercise illustrated that many childcare providers (92%) were aware of the CHVs, and several mentioned that they already visit the centers to check the children's health. Similarly, all but one of the 22 CHVs completing the questionnaire reported supporting childcare providers and 62% (13 CHVs) reported visiting weekly (Table 6).

“*When the CHV's are making their rounds as part of their check on children's health, often you find them going to daycares as they know they will get children in the daycare centers. So, they have to visit me and check them and if they find out the child has poor health, they tell me what to do.”* (Parent K13, female, married and living with husband, daily wage earner, Korogocho).

The idea of using the CHVs to deliver the intervention was discussed with county and sub-county officials and considered by all stakeholders during the co-design workshops. Ultimately, it was agreed that CHVs would be the most appropriate members of the community health team to be trained to deliver the intervention. This was reiterated in the workshop where participants felt that as CHVs were already visiting households, and many were already including childcare centers in their rounds, this would not be an extra burden.

“*It will not be too much work because the day care centres are in the community so the CHVs can work in them as they do their other duties. Some of the childcares are part of homes and the CHVs do household visits so for childcares within the household environment, it will be easy to visit. The CHVs live in the community, and they know where the childcare centres are. If the CHVs are facilitated, they can do the work well. However, they need to be trained on what they should look out for.”* (Workshop 3, participant from Nairobi Metropolitan Services).

The CHVs and their CHA supervisor agreed that supporting child health and development in the childcare centers was within the CHV's remit. However, they identified resources, training, and systems adaptations that would be required to support implementation. The questionnaire results concurred with the 22 CHVs and showed that they felt motivated to support the centers, with 16 feeling strongly motivated. The most common barriers identified by the CHVs were their lack of tools and resources (68%), lack of training (55%), and perceptions of low interest among center providers (45%) (see Table 6).

“*CHVs will be able to support the project but for them to do so, they will need training on the project. They will also need stipends, referral books, field supplies such as gum boots, raincoats, bags and umbrellas; they will also need hand washing equipment to place in the day care centres; they will also need certificates and badges from the project/organization.”* (Participant in Workshop 4 with CHAs and CHVs working in Viwandani).

“*The public health reporting tool does not have a component to capture childcare centres so this can be added. Currently there are no data on childcare centres.”* (Participant in Workshop 1 with sub-county local government teams).

Sub-county and community health teams were quick to identify the potential of the intervention to improve the health and early childhood development outcomes of children, particularly through the early identification of disabilities such as hearing impairment and autism, increased awareness of child rights and action to protect children from abuse (Workshop 1 sub-county local government), and the potential to increase uptake of immunization, vitamin A, and deworming programs and improve providers skills to assess sick children and know what to do in case of a health emergency (Workshop 2 Community Health team, Workshop 5 Sub-county local government). They also highlighted challenges in implementing the intervention, particularly due to the instability within informal settlements with both families and childcare centers frequently moving locations due to evictions and the continual struggles for daily survival and economic opportunities. Some CHVs felt that training on income-generating activities for childcare providers could help to overcome these difficulties, and all agreed that some component of training to support providers to run their centers as viable businesses would be an important component of the intervention.

During workshop 3 with representatives from Nairobi Metropolitan Services (NMS), the challenges of operationalizing the current Nairobi City County Childcare Facilities Act 2017 were highlighted, in particular the registration of childcare centers within informal settlements. The Act specifies a minimum standard for facilities, staffing, and training for childcare centers, and the center must meet these standards and pay a registration fee.

“*Some of these centres are not registered and therefore no one has gone to assess the quality of service they provide because no one even knows where they are because they have never been mapped.”* (Participant, Workshop 3 Nairobi Metropolitan Services).

## Intervention design

The co-design workshops and rapid analysis of the qualitative data informed the development of a list of topics to be covered in the training. The process of close engagement with childcare providers, CHVs, and parents built the teams' understanding of the operational context and how the intervention should be delivered. It was evident from the interviews that there was diversity in the levels of experience and knowledge of the childcare providers. Their need to work long hours providing care, particularly for the home-based center providers, was also evident.

**Figure 4:**
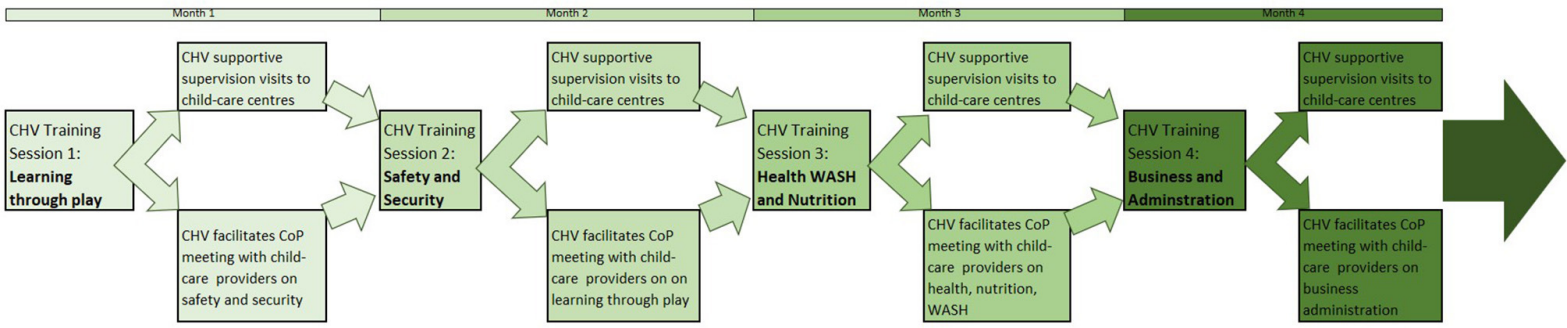
Childcare communities of practice model.

**Figure 5:**
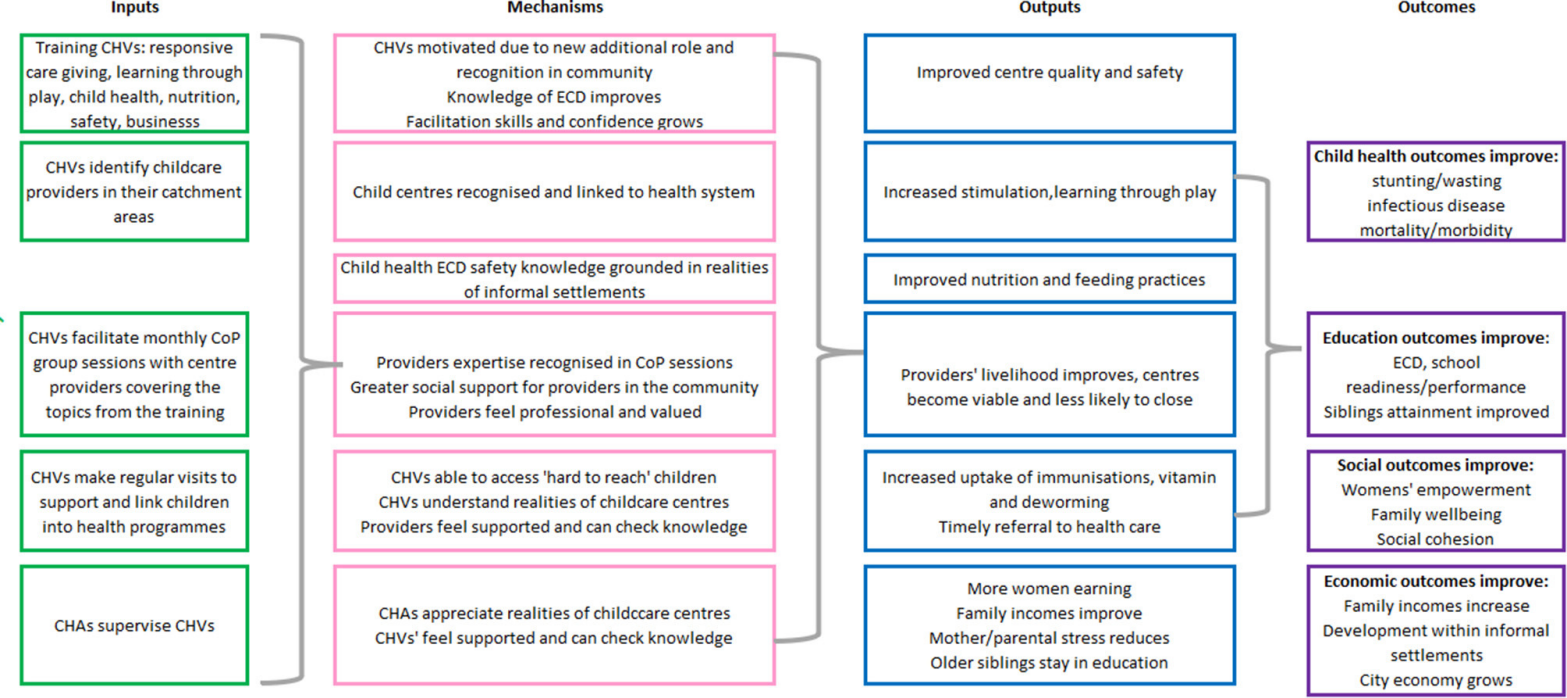
Theory of change.

Deliberation on these factors within the workshops (particularly workshops 6, 7, and 8) led to many design decisions. First, a CoP approach (38) which allows providers to share experiences and approaches and to apply good practice to the context of informal settlements would be more appropriate than a didactic training course. Second, the challenging and unstable economic conditions of the informal settlement and limited business experience of the providers, particularly the home-based providers, would need to be a component of any support to improve the quality and functioning of the centers. Third, CHVs would be well placed to facilitate these CoP meetings due to their existing role in the community, but they required further training particularly in areas of early childhood development, child safety, and business management, with top-up training on child health, hygiene, and nutrition. Fourth, given the hours worked by childcare providers, particularly the home-based centers, CoP meetings should be held at the weekend with compensation paid to providers to enable them to pay a back-up career during their absence. Fifth, support to individual centers was required between the CoP session, and CHVs were well-placed to include these supportive supervision sessions within their regular household rounds. The details of the intervention are provided in Table 7 below, using the headings provided by the Template for Intervention Description and Replication (TiDieR) (49) to describe each aspect. The implementation flow of the intervention over the 4-month feasibility study is provided in Figure 4 and the theory of change is shown in Figure 5.

**Table 7:** Detailed description of the intervention following the TiDieR checklist.

1. Brief name:	Childcare communities of practice	
2. Why	To improve the knowledge, skills, and practice of childcare providers to improve the quality of childcare in informal settlements for healthy child development	
3. What:	Materials	• Simple quality assessment tool • Training slides to run 4 modules: (1) Learning through play; (2) child protection and safety; (3) Nutrition, health, and hygiene; and (4) Business administration. Each module took 2 half-days of training and an estimate of 12 h, except the 3rd module on nutrition which took three half-days and about 18 h. • Trainer's notes: Detailed notes to assist the trainer to deliver the content of the four sessions. • Handouts: Short notes, posters, and other resources to be given to the community volunteers to share with center providers during the CoP sessions and childcare center visits. • Flip charts, marker pens, masking tapes, notebooks, and pens for use during training and the CoP sessions. • For practical lessons during the training and the CoP sessions, participants (CHVs and childcare providers) brought locally available recycled materials (e.g., toilet paper rolls, bottle tops, used boxes, and plastic bottles) to make toys and early learning aids.
4.	Procedures	• Provide an initial induction session to the Community volunteers about the CoP program and how to identify the child-care centers within the selected communities and conduct a simple quality assessment using the quality assessment tool (approx. half day) • Community volunteers identify centers and conduct quality assessment. • Training of community volunteers, 4 modules ~6 days spread over 4–6 months: (1) Learning through play; (2) child protection and safety; (3) Nutrition, health, and hygiene; and (4) Business administration. Each module took two half-days of training and ~12 h in total to deliver except the module on nutrition, health, and hygiene which took three-half days and approximately 18 h to deliver. • Community volunteers facilitate monthly CoP groups of ~8 childcare providers at a time convenient for them on the topics covered in their training. • Community volunteers visit the childcare centers (covering all CoP group members in a month) to support the implementation of new skills/practice and to monitor quality using the simple assessment tool. • Supervisors (CHAs) meet volunteers regularly (as per their routine supervision) and accompany them on childcare visits or during CoP group sessions as needed.
5. Who	Community volunteers (e.g., CHVs)	• Community workers or volunteers with an interest and minimal training in child health and development would be appropriate to deliver the intervention. They need to have time available to visit childcare centers and run monthly group CoP sessions within their neighborhood. CHVs receive a stipend to cover the expenses of their work.
	Supervisors of community volunteers	• Within our intervention, the CHAs supervised the CHVs through their routine meetings and supervision structures and provided training for CHVs. The CHAs hold at least a diploma in any health discipline and they are the primary supervisors of the CHVs. They will provide supportive supervision to the CHVs during the CoP sessions and assessment visits.
	Local experts on child protection ECD, health, and nutrition	• To supplement the knowledge/skills of the main supervisors (e.g., CHAs) during the training. Experts will require a good understanding of the context to be able to ensure topic knowledge is applied and relevant.
6. How		• Community volunteers facilitate monthly face-to-face CoP sessions to facilitate trained center providers to share knowledge and experience on the four module areas. Sessions last several hours depending on provider availability. • Visits from the community volunteers to the childcare centers are in-person, informal, and supportive.
7. Where		• Training can be held in a suitable community venue. • CoP sessions can be held in childcare centers when no children are using the center. Hosting CoP sessions can rotate between group members. This helps to ensure discussions are grounded in the realities of the center environment and facilities.
8. When and how much		• The total intervention can be delivered in 4 months, however, 6 months may be more appropriate, with the 6 days training of community volunteers spread over the period. This ensures the CoP sessions on the same topic are held soon after the training. • Monthly CoP sessions last 1 or 2 h, or longer depending on the interest of the members. • Community volunteers should visit all center providers from the group (~8) during the month between CoP sessions (~2 centers visited each week).
9. Tailoring and implementation		• The changes made to the intervention during delivery will be described in a subsequent study.

## Discussion

Our results show the presence of a high number of childcare centers in Korogocho and Viwandani which is an indication of the growing demand for childcare services in these informal settlements. The qualitative and quantitative findings highlight the challenges facing these centers within every domain of the nurturing care framework.

Our findings reflect those of the limited number of other studies that have explored the quality of childcare in informal settlements (50). Other studies within the African context have assessed quality (51) and the impacts of types of preschool environment on childhood development (52), finding the importance of play materials, infrastructure, and training of providers on ECD. Previous work in informal settlements in Kenya found an average child-to-adult ratio of 1:22, limited nutrition, poor sanitation and facilities, and untrained caregivers (53). The informal home-based centers were often identified by parents and community health workers and volunteers as having the most limited facilities, environment, and poor practice in ECD and health. Similar quality issues have been reported in other studies that have examined the provision of childcare within similar urban settlements in Africa (54). The ability of these centers to provide “nurturing care” (55) is severely limited, leaving parents anxious about the quality of childcare and undermining child health and development.

The Nurturing Care Framework (56) provided a valuable framework for identifying appropriate content for the intervention. We identified significant gaps in center resources, practices, and knowledge of providers in all areas of the framework. This was the case across all types of providers, particularly the small informal home-based and center-based providers. However, the inputs for all participant groups (center-providers, health workers and volunteers, parents, and decision-makers) through the co-design workshops were key in determining the focus and depth of the topics within each domain of the NCF. The decision to follow a CoP approach was reinforced by the findings from parents and childcare providers on the diversity of approaches and the considerable strengths and levels of experience among the providers, with clear benefit to be achieved from sharing these experiences through group CoP sessions. One element emerged that is not emphasized within the NCF and not originally considered in depth by the team was the importance of supporting center management. The challenges of childcare providers attempting to balance limited incomes with high expenditures, particularly following the economic impacts of the COVID lockdowns were very evident in the qualitative interviews and co-design workshops in our study. Previous studies have noted an association between center management capacity and center quality (51), yet management and leadership are rarely considered in the assessment of the quality of childcare centers (57).

There is limited evidence on how to support childcare centers, particularly smaller home-based informal center to improve healthy childhood development, particularly in LMICs (5, 58). While Kenya has a strong track record in ECD policy, starting with the ECD Policy Framework of 2006 and encouraging recent attempts to bring multiple sectors together to strengthen the response to ECD (46), there is clearly a wide gap between policy and practice. This is particularly the case in Nairobi where the County government leads policy provision in the country with the Nairobi City County Childcare Facilities Act 2017 which clearly specifies standards for childcare services. We found that most informal childcare centers were unregistered and only received *ad hoc* visits from community health teams. Our study highlights the need to ensure that policies and strategic plans reflect the realities facing the growing number of informal home-based and small private centers and can realistically be implemented within low-income neighborhoods. Implementing and scaling up the registration process linked to achievable standards, combined with support from CHVs and the community health teams, has the potential to bridge this policy and practice gap.

Our intervention development process has much in common with the characteristics of co-design described by Vargas et al. (59) where multiple stakeholders actively collaborate to design solutions to prespecified problems. This contrasts with a process of “co-creation” where diverse stakeholders are deeply involved in the process of understanding problems and evaluating solutions (59), a process with similarities to participatory action research (60). Within the context of our intervention development process, we took as our starting point the consensus and evidence base underpinning the Nurturing Care Framework (56) and the need to ensure the intervention could be delivered sustainably with the existing system due to the lack of any additional resources. Our co-design process focused on developing an intervention within these parameters was preferable.

We were cognizant of the need to ensure that the intervention was well-integrated into existing systems and accepted by parents and childcare providers. We were particularly conscious of the need to ensure CHVs were not overburdened and received sufficient training and supportive supervision as these factors have been identified as undermining their wellbeing and willingness to continue their vital work (61, 62). Our qualitative findings and the discussions in the co-design workshops highlighted that some CHVs were already visiting childcare providers but in an *ad hoc* manner and with limited knowledge of how to support the centers. The lack of organized support, training, and regular contact of childcare centers with the community health services was an important finding and influenced co-design decisions to focus the intervention on the informal, home-based childcare centers. Agreement across community health teams, and local and national government of the potential contribution of CHVs in providing structured support to childcare centers, was a major step in the co-design process. Recent government plans to provide a stipend of KES 2,000/month (~US$20) for CHVs and Nairobi county's commitment to their community health bill bodes well for improved functioning and sustainable functioning of the CHV program (63).

We developed a highly participatory process to co-design ensuring there were multiple ways to hear the voices of the different tiers of government, CHVs, their supervisors, as well as a range of childcare providers and parents. By using qualitative interviews as well as the co-design workshop and knowledge, attitudes, and practices questionnaires, we felt able to shape the intervention to fit well into the health system and community context. In addition, we were very aware of the important role played by NGOs in filling many of the service gaps in informal settlements. Our partners in this study, Kidogo (see https://www.kidogo.co/, co-author MK) provided detailed guidance and resources. However, scaling up and sustaining initiatives to improve the quality of childcare is vital in the context of rapid urbanization (63). Our study will also publish findings on the feasibility of implementation. However, further research is needed to assess the sustainability and effectiveness of such interventions to improve child health and ECD and contribute to wider outcomes such as women's participation in the workforce and household income and wellbeing.

## Strengths and limitations

This study had some limitations as the COVID-19 pandemic hit the country in March 2020 when study implementation was just taking-off. This meant that co-design workshops and baseline qualitative interviews were conducted virtually and elements of the co-design process, which were planned as highly participatory group events with the use of video, had to be simplified to be conducted virtually or in accordance with social distancing requirements. The pandemic period also resulted in the closure of some childcare centers. This means that our estimates of the number of centers and the number of children using the centers may not reflect non-pandemic conditions.

Few studies have explored childcare conditions in informal settlements. Our long-term engagement using both quantitative and qualitative methods as well as the co-design process enabled deeper insights into the assets and challenges facing different types of childcare providers in informal settlements. An additional strength of our study is the co-design approach which enabled the complete engagement of national and county governments, parents, childcare providers, and community health teams. The engagement of these multiple stakeholders was a prerequisite to the design of an intervention embedded within government systems to enable potential scale-up and sustainable delivery.

## Conclusion

Implementing a co-design process embedded within existing community health systems and drawing on the lived experiences of childcare providers and parents in informal settlements facilitated the development of an intervention with the potential to be scalable and sustainable. Such interventions are urgently required as the number of home-based and small center-based informal childcare centers are growing rapidly to meet demand; yet, they receive little support to improve quality and are largely unregulated. Given the context of informal settlements, support to strengthen management within the centers is vital in addition to the core domains of WHO's Nurturing Care Framework. Further research on the effectiveness and sustainability of support to private and informal childcare centers in the context of low-income urban neighborhoods is needed.

## Data availability statement

The original contributions presented in the study are included in the article/Supplementary material, further inquiries can be directed to the corresponding author.

## Ethics statement

The studies involving human participants were reviewed and approved by Amref Ethics and Scientific Review Committee. The patients/participants provided their written informed consent to participate in this study.

## Author contributions

LO led in writing, review and editing the manuscript and participated in the project administration, supervision, data curation, and formal analysis. HE led the acquisition of funding, conceptualization, investigation, methodology, contributed to project administration, formal analysis, and writing of the manuscript. MA-O participated in project administration, supervision, data analysis, and writing of the manuscript. MK participated in project administration and writing of the manuscript. PA participated in the project administration, data curation, formal analysis, and writing of the manuscript. KO participated in the conceptualization, investigation and methodology of the study, project administration, data curation, formal analysis, and writing of the manuscript. PK-W participated in the conceptualization, investigation and methodology, project administration, data curation and visualization, and writing of the manuscript. EK-M contributed to the design, supported the administration of the project, and writing of the manuscript. NL led formal data analysis and curation, participated in project administration, and writing of the manuscript. MN co-led the acquisition of funding, conceptualization, investigation, methodology of the study, supervision, data analysis, and writing the manuscript. All authors participated in the writing, reviewing, and editing of the final version of the manuscript. All authors contributed to the article and approved the submitted version.
