# Supplementary material for: Developing an intervention to improve the quality of childcare centers in resource-poor urban settings: a mixed methods study in Nairobi, Kenya
Source: Front Public Health. 2023 Jul 17;11:1195460. doi: 10.3389/fpubh.2023.1195460 (PMC10387541; doi:10.3389/fpubh.2023.1195460)
Supplement: Supplementary file 1 [file Table_1.DOCX]

### ****AFRICAN POPULATION AND HEALTH RESEARCH CENTER****

#### KAP - Center Providers

| \|  \|  \| generated_note_name_43 \| \| --- \| --- \| --- \| | ****Section1: Centre profile**** |  |
| --- | --- | --- | --- | --- | --- |
| \|  \|  \| interview_date (required) \| \| --- \| --- \| --- \| | i. **Date** of interview  DON’T CHANGE THIS ENTRY |  |
| \|  \|  \| interviewer (required) \| \| --- \| --- \| --- \| | ii. **Name** of interviewer |  |
| \|  \|  \| start_time (required) \| \| --- \| --- \| --- \| | iii. Start **time** |  |
| \|  \|  \| q1 (required) \| \| --- \| --- \| --- \| | 1. Child care centre ID: |  |
| \|  \|  \| q2 (required) \| \| --- \| --- \| --- \| | 2. Center **provider ID** |  |
| \|  \|  \| cp_3 (required) \| \| --- \| --- \| --- \| | 3. Center provider age (years) |  |
| \|  \|  \| cp_4 (required) \| \| --- \| --- \| --- \| | 4. Center provider sex | \|  \| 1 \| Male \| \| --- \| --- \| --- \| \|  \| 2 \| Female \| |
| \|  \|  \| cp_5 (required) \| \| --- \| --- \| --- \| | 5. Center provider highest education level: | \|  \| 1 \| None \| \| --- \| --- \| --- \| \|  \| 2 \| Primary \| \|  \| 3 \| Secondary \| \|  \| 4 \| Tertiary(including University) \| |
| \|  \|  \| q3 (required) \| \| --- \| --- \| --- \| | 3. **Location** of centre | \|  \| 1 \| Korogocho \| \| --- \| --- \| --- \| \|  \| 2 \| Viwandani \| |
| \|  \|  \| q4 (required) \| \| --- \| --- \| --- \| | 4. **Type** of centre | \|  \| 1 \| Home based \| \| --- \| --- \| --- \| \|  \| 2 \| Centre based (up-to age 6) \| \|  \| 3 \| Attached to primary school \| \|  \| 4 \| church based \| \|  \| 96 \| Other type (specify) \| |
| \|  \| access_electronic_alot > sec1_grp2_header \| \| --- \| --- \| | | |
| \|  \|  \| generated_note_name_73 \| \| --- \| --- \| --- \| | Center provider ID: **CP** |  |
| \|  \|  \| q4_os (required) \| \| --- \| --- \| --- \| | 4. Other type **(specify)** |  |
| \|  \|  \| q5 (required) \| \| --- \| --- \| --- \| | 5. **Total children enrolled** in the center |  |
| \|  \|  \| q6 (required) \| \| --- \| --- \| --- \| | 6. **Youngest** child’s age: |  |
| \|  \|  \| q7 (required) \| \| --- \| --- \| --- \| | 7. **Oldest** child’s age: |  |
| \|  \|  \| q8 (required) \| \| --- \| --- \| --- \| | 8. Total children enrolled **below 3 yrs** |  |
| \|  \|  \| q9 (required) \| \| --- \| --- \| --- \| | 9. Number of **caregivers** in the centre |  |
| \|  \|  \| q10 (required) \| \| --- \| --- \| --- \| | 10. Has the Center provider received (currently or previously) **training in looking after young children and early childhood development** ? | \|  \| 1 \| Yes \| \| --- \| --- \| --- \| \|  \| 2 \| No \| |
| \|  \| q11b (required) \| \| --- \| --- \| | 11b. Specify **who received** the training |  |
| \|  \| q11c (required) \| \| --- \| --- \| | 11c. Specify **what type** of training (topic) |  |
| \|  \| q11d (required) \| \| --- \| --- \| | 11d. **Who provided** the training (provided by whom) |  |
| \|  \| access_electronic_alot > centers_operation__grp_i \| \| --- \| --- \| | | |
| \|  \|  \| generated_note_name_99 \| \| --- \| --- \| --- \| | ****Centre operation (business management)**** |  |
| \|  \|  \| opening_time (required) \| \| --- \| --- \| --- \| | 12. **Opening** time |  |
| \|  \|  \| closing_time (required) \| \| --- \| --- \| --- \| | 13. **Closing** time |  |
| \|  \|  \| centers_operation__grp_i_options \| \| --- \| --- \| --- \| | OPTIONS | \|  \| 1 \| Yes \| \| --- \| --- \| --- \| \|  \| 2 \| No \| |
| \|  \|  \| q14 (required) \| \| --- \| --- \| --- \| | 14 Do they open on **Saturday** ? | \|  \| 1 \| Yes \| \| --- \| --- \| --- \| \|  \| 2 \| No \| |
| \|  \| q14_os (required) \| \| --- \| --- \| | 14 If yes, clarify whether it is regular or on special request | \|  \| 1 \| Regular \| \| --- \| --- \| --- \| \|  \| 2 \| Special request \| |
| \|  \| q15 (required) \| \| --- \| --- \| | 15 Do they open on **Sunday** ? | \|  \| 1 \| Yes \| \| --- \| --- \| --- \| \|  \| 2 \| No \| |
| \|  \| q15_os (required) \| \| --- \| --- \| | 15 If yes, clarify whether it is regular or on special request | \|  \| 1 \| Regular \| \| --- \| --- \| --- \| \|  \| 2 \| Special request \| |
| \|  \| access_electronic_alot > knw_busines_mgt_grp_ii \| \| --- \| --- \| | | |
| \|  \|  \| generated_note_name_120 \| \| --- \| --- \| --- \| | ****Knowledge questions on Business management**** |  |
| \|  \|  \| generated_note_name_122 \| \| --- \| --- \| --- \| | 1 - Agree completely  2 - Agree a little  3 - Disagree a little  4 - Disagree completely |  |
| \|  \|  \| knw_busines_mgt_grp_ii_options \| \| --- \| --- \| --- \| | OPTIONS | \|  \| 1 \| 1 \| \| --- \| --- \| --- \| \|  \| 2 \| 2 \| \|  \| 3 \| 3 \| \|  \| 4 \| 4 \| |
| \|  \|  \| q16 (required) \| \| --- \| --- \| --- \| | 16. It is important to develop the budget for the day-care centre at the beginning of the term | \|  \| 1 \| 1 \| \| --- \| --- \| --- \| \|  \| 2 \| 2 \| \|  \| 3 \| 3 \| \|  \| 4 \| 4 \| |
| \|  \|  \| q17 (required) \| \| --- \| --- \| --- \| | 17. It is important to record which parent has paid | \|  \| 1 \| 1 \| \| --- \| --- \| --- \| \|  \| 2 \| 2 \| \|  \| 3 \| 3 \| \|  \| 4 \| 4 \| |
| \|  \|  \| q18 (required) \| \| --- \| --- \| --- \| | 18. It is important to record how much each parent has paid | \|  \| 1 \| 1 \| \| --- \| --- \| --- \| \|  \| 2 \| 2 \| \|  \| 3 \| 3 \| \|  \| 4 \| 4 \| |
| \|  \|  \| q19 (required) \| \| --- \| --- \| --- \| | 19. Do you/center owner **track the income and expenses** made in your centre? | \|  \| 1 \| Yes \| \| --- \| --- \| --- \| \|  \| 2 \| No \| |
| \|  \| q20 (required) \| \| --- \| --- \| | 20 If yes, **how do you** track them? |  |
| \|  \| q21 (required) \| \| --- \| --- \| | 21. Do you make or **prepare a budget** for your center at the beginning of the week/month? | \|  \| 1 \| Yes \| \| --- \| --- \| --- \| \|  \| 2 \| No \| |
| \|  \| q22 (required) \| \| --- \| --- \| | 22. Do you have the centre policy on pricing and opening times for the centre? (centre name, operating hrs, fees, caregiver contacts).  Ask the center provider if it is not visible | \|  \| 1 \| Yes \| \| --- \| --- \| --- \| \|  \| 2 \| No \| |
| \|  \| q23 (required) \| \| --- \| --- \| | 23 Which **social media** do you (center provider) use?  Tick all that apply | \|  \| 1 \| WhatsApp \| \| --- \| --- \| --- \| \|  \| 2 \| Facebook \| \|  \| 3 \| Instagram \| \|  \| 4 \| Twitter \| \|  \| 99 \| None \| |
| \|  \| q24 (required) \| \| --- \| --- \| | 24 Do you **track/keep accounts** for your center showing income and expenditure? | \|  \| 1 \| Yes \| \| --- \| --- \| --- \| \|  \| 2 \| No \| |
| \|  \| q25 (required) \| \| --- \| --- \| | 25 Do you **track attendance** daily? | \|  \| 1 \| Yes \| \| --- \| --- \| --- \| \|  \| 2 \| No \| |
| \|  \| q26 (required) \| \| --- \| --- \| | 26 Is the **business license** posted? | \|  \| 1 \| Yes \| \| --- \| --- \| --- \| \|  \| 2 \| No \| |
| \|  \| q27 (required) \| \| --- \| --- \| | 27 Do you have an **attendance register** showing how many children come to your centre per day? | \|  \| 1 \| Yes \| \| --- \| --- \| --- \| \|  \| 2 \| No \| |
| \|  \| access_electronic_alot > knw_safety_grp_i \| \| --- \| --- \| | | |
| \|  \|  \| generated_note_name_155 \| \| --- \| --- \| --- \| | ****Knowledge Questions on Safety**** |  |
| \|  \|  \| generated_note_name_157 \| \| --- \| --- \| --- \| | **Provision of safe and stimulating environment** |  |
| \|  \|  \| generated_note_name_159 \| \| --- \| --- \| --- \| | 1 - Agree completely  2 - Agree a little  3 - Disagree a little  4 - Disagree completely |  |
| \|  \|  \| knw_safety_grp_i_options \| \| --- \| --- \| --- \| | OPTIONS | \|  \| 1 \| 1 \| \| --- \| --- \| --- \| \|  \| 2 \| 2 \| \|  \| 3 \| 3 \| \|  \| 4 \| 4 \| |
| \|  \|  \| q28 (required) \| \| --- \| --- \| --- \| | 28. It is important to always ensure a safe environment for the children | \|  \| 1 \| 1 \| \| --- \| --- \| --- \| \|  \| 2 \| 2 \| \|  \| 3 \| 3 \| \|  \| 4 \| 4 \| |
| \|  \|  \| q29 (required) \| \| --- \| --- \| --- \| | 29 It is important for a caregiver or other people to **keep a child in visual range** and to look at him/her often | \|  \| 1 \| 1 \| \| --- \| --- \| --- \| \|  \| 2 \| 2 \| \|  \| 3 \| 3 \| \|  \| 4 \| 4 \| |
| \|  \|  \| generated_note_name_167 \| \| --- \| --- \| --- \| | Give three examples of **common hazards** in the day care center |  |
| \|  \|  \| q31_haz1 (required) \| \| --- \| --- \| --- \| | 31 (i) Hazard 1 |  |
| \|  \|  \| q31_haz2 (required) \| \| --- \| --- \| --- \| | 31 (ii) Hazard 2 |  |
| \|  \|  \| q31_haz3 (required) \| \| --- \| --- \| --- \| | 31 (iii) Hazard 3 |  |
| \|  \|  \| generated_note_name_176 \| \| --- \| --- \| --- \| | 1 - Agree completely  2 - Agree a little  3 - Disagree a little  4 - Disagree completely |  |
| \|  \|  \| q30_options \| \| --- \| --- \| --- \| | OPTIONS | \|  \| 1 \| 1 \| \| --- \| --- \| --- \| \|  \| 2 \| 2 \| \|  \| 3 \| 3 \| \|  \| 4 \| 4 \| |
| \|  \|  \| q30 (required) \| \| --- \| --- \| --- \| | 30. Center providers should constantly **watch out** for and remove any potentially **hazardous materials** (e.g. sharps, fire, ditches, live wires, collapsing walls, hot water, medicines, small beads/beans) | \|  \| 1 \| 1 \| \| --- \| --- \| --- \| \|  \| 2 \| 2 \| \|  \| 3 \| 3 \| \|  \| 4 \| 4 \| |
| \|  \|  \| safety_comment \| \| --- \| --- \| --- \| | **Comment** |  |
| \|  \| access_electronic_alot > responsive_caregiving_grp \| \| --- \| --- \| | | |
| \|  \|  \| generated_note_name_188 \| \| --- \| --- \| --- \| | ****Knowledge questions on responsive caregiving/discipline**** |  |
| \|  \|  \| generated_note_name_190 \| \| --- \| --- \| --- \| | **Responsive caregiving /positive discipline/child abuse** |  |
| \|  \|  \| q32 (required) \| \| --- \| --- \| --- \| | 32 Children must be **handled harshly** for them to develop better. | \|  \| 1 \| Always \| \| --- \| --- \| --- \| \|  \| 2 \| Sometimes \| \|  \| 3 \| Never \| |
| \|  \|  \| q33 (required) \| \| --- \| --- \| --- \| | 33 Which **method** do you use most often to deal with children when they **misbehave** ?  (tick only one option) | \|  \| 1 \| Physical punishment \| \| --- \| --- \| --- \| \|  \| 2 \| Verbal punishment \| \|  \| 3 \| Distract child with another activity \| \|  \| 4 \| Explains wrong deeds to child calmly \| |
| \|  \|  \| q34 (required) \| \| --- \| --- \| --- \| | 34. In the past 2 weeks, how many **times** have children been physically **punished in the centre** ?  (pinch/slap/spanked ) | \|  \| 1 \| Daily \| \| --- \| --- \| --- \| \|  \| 2 \| Once to twice in a week \| \|  \| 3 \| Never \| |
| \|  \|  \| res_cgvn_comment \| \| --- \| --- \| --- \| | **Comment** |  |
| \|  \| access_electronic_alot > learning_play_grp \| \| --- \| --- \| | | |
| \|  \|  \| generated_note_name_207 \| \| --- \| --- \| --- \| | ****Learning through play**** |  |
| \|  \|  \| generated_note_name_209 \| \| --- \| --- \| --- \| | 1 - Agree completely  2 - Agree a little  3 - Disagree a little  4 - Disagree completely |  |
| \|  \|  \| learning_play_grp_options \| \| --- \| --- \| --- \| | OPTIONS | \|  \| 1 \| 1 \| \| --- \| --- \| --- \| \|  \| 2 \| 2 \| \|  \| 3 \| 3 \| \|  \| 4 \| 4 \| |
| \|  \|  \| q35 (required) \| \| --- \| --- \| --- \| | 35 It is important for children to play? | \|  \| 1 \| 1 \| \| --- \| --- \| --- \| \|  \| 2 \| 2 \| \|  \| 3 \| 3 \| \|  \| 4 \| 4 \| |
| \|  \|  \| q36 (required) \| \| --- \| --- \| --- \| | 36 Which of the following **activities do you do in a typical day** with the children  (Tick all that applies) | \|  \| 1 \| Play with toys \| \| --- \| --- \| --- \| \|  \| 2 \| Singing \| \|  \| 3 \| Dancing \| \|  \| 4 \| Movement/games \| \|  \| 5 \| Storytelling/reading \| \|  \| 6 \| Counting \| \|  \| 7 \| Drawing/making things \| \|  \| 8 \| Playing with water/sand \| |
| \|  \|  \| learning_play_q1p3_p4_options \| \| --- \| --- \| --- \| | OPTIONS | \|  \| 1 \| Yes \| \| --- \| --- \| --- \| \|  \| 2 \| No \| |
| \|  \|  \| q37 (required) \| \| --- \| --- \| --- \| | 37. Does each child get an opportunity to play with toy or something | \|  \| 1 \| Yes \| \| --- \| --- \| --- \| \|  \| 2 \| No \| |
| \|  \|  \| q38 (required) \| \| --- \| --- \| --- \| | 38. Do you post the Children’s work on the wall | \|  \| 1 \| Yes \| \| --- \| --- \| --- \| \|  \| 2 \| No \| |
| \|  \|  \| learning_play_comment \| \| --- \| --- \| --- \| | **Comment** |  |
| \|  \| access_electronic_alot > health_grp \| \| --- \| --- \| | | |
| \|  \|  \| generated_note_name_232 \| \| --- \| --- \| --- \| | ****Knowledge questions on health**** |  |
| \|  \|  \| generated_note_name_234 \| \| --- \| --- \| --- \| | 1 - Agree completely  2 - Agree a little  3 - Disagree a little  4 - Disagree completely |  |
| \|  \|  \| health_grp_options \| \| --- \| --- \| --- \| | OPTIONS | \|  \| 1 \| 1 \| \| --- \| --- \| --- \| \|  \| 2 \| 2 \| \|  \| 3 \| 3 \| \|  \| 4 \| 4 \| |
| \|  \|  \| q39 (required) \| \| --- \| --- \| --- \| | 39. It is important to always take a child’s temperature on arrival at the day care | \|  \| 1 \| 1 \| \| --- \| --- \| --- \| \|  \| 2 \| 2 \| \|  \| 3 \| 3 \| \|  \| 4 \| 4 \| |
| \|  \|  \| generated_note_name_240 \| \| --- \| --- \| --- \| | 40. Mention three common examples of **possible emergencies** that may occur to children while in a day care center (any 3 correct answer=score 1) |  |
| \|  \|  \| q40_emergency1 (required) \| \| --- \| --- \| --- \| | 40 (i) Emergency 1 |  |
| \|  \|  \| q40_emergency2 (required) \| \| --- \| --- \| --- \| | 40 (ii) Emergency 2 |  |
| \|  \|  \| q40_emergency3 (required) \| \| --- \| --- \| --- \| | 40 (iii) Emergency 3 |  |
| \|  \|  \| q41_42_options \| \| --- \| --- \| --- \| | OPTIONS | \|  \| 1 \| Yes \| \| --- \| --- \| --- \| \|  \| 2 \| No \| |
| \|  \|  \| q41 (required) \| \| --- \| --- \| --- \| | 41. Conduct daily health checks? | \|  \| 1 \| Yes \| \| --- \| --- \| --- \| \|  \| 2 \| No \| |
| \|  \|  \| q42 (required) \| \| --- \| --- \| --- \| | 42. Do you understand what to do if a child is sick? | \|  \| 1 \| Yes \| \| --- \| --- \| --- \| \|  \| 2 \| No \| |
| \|  \| q43 (required) \| \| --- \| --- \| | 43. What would you do? |  |
| \|  \| q44 (required) \| \| --- \| --- \| | 44. Do you have a **first aid kit** ?  (Request to look at the contents for the kit) | \|  \| 1 \| Yes \| \| --- \| --- \| --- \| \|  \| 2 \| No \| |
| \|  \| q45 (required) \| \| --- \| --- \| | 45. Do you know **how to use** the first aid kit? | \|  \| 1 \| Yes \| \| --- \| --- \| --- \| \|  \| 2 \| No \| |
| \|  \| q46 (required) \| \| --- \| --- \| | 46. Do you know if the children you care for have been **immunised** ? | \|  \| 1 \| Yes for all \| \| --- \| --- \| --- \| \|  \| 2 \| Yes for some \| \|  \| 3 \| No \| |
| \|  \| q47 (required) \| \| --- \| --- \| | 47. (specify **how many**………) |  |
| \|  \| access_electronic_alot > health_grp_ii \| \| --- \| --- \| | | |
| \|  \|  \| generated_note_name_270 \| \| --- \| --- \| --- \| | Can you tell me which **vaccinations** children should have and when?  **Interviewer confirms if answer given by center provider is correct or wrong** : |  |
| \|  \|  \| q48_52_options \| \| --- \| --- \| --- \| | OPTIONS | \|  \| 1 \| Correct \| \| --- \| --- \| --- \| \|  \| 2 \| Wrong \| |
| \|  \|  \| q48 (required) \| \| --- \| --- \| --- \| | 48. Birth (BCG & Polio 0) | \|  \| 1 \| Correct \| \| --- \| --- \| --- \| \|  \| 2 \| Wrong \| |
| \|  \|  \| q49 (required) \| \| --- \| --- \| --- \| | 49. six weeks (DPT and Polio 1) | \|  \| 1 \| Correct \| \| --- \| --- \| --- \| \|  \| 2 \| Wrong \| |
| \|  \|  \| q50 (required) \| \| --- \| --- \| --- \| | 50. Ten weeks(DPT2 and Polio 2) | \|  \| 1 \| Correct \| \| --- \| --- \| --- \| \|  \| 2 \| Wrong \| |
| \|  \|  \| q51 (required) \| \| --- \| --- \| --- \| | 51. Fourteen weeks (DPT3 and Polio 3) | \|  \| 1 \| Correct \| \| --- \| --- \| --- \| \|  \| 2 \| Wrong \| |
| \|  \|  \| q52 (required) \| \| --- \| --- \| --- \| | 52. Nine months (measles) | \|  \| 1 \| Correct \| \| --- \| --- \| --- \| \|  \| 2 \| Wrong \| |
| \|  \|  \| generated_note_name_284 \| \| --- \| --- \| --- \| | (NB interviewer will have to know the immunisation schedule and compare their answer to tick correct or wrong)  *Immunization records kept on file* |  |
| \|  \|  \| health_grp_comment \| \| --- \| --- \| --- \| | **Comment** |  |
| \|  \| access_electronic_alot > nutrition_grp \| \| --- \| --- \| | | |
| \|  \|  \| generated_note_name_292 \| \| --- \| --- \| --- \| | ****Nutrition**** |  |
| \|  \|  \| generated_note_name_296 \| \| --- \| --- \| --- \| | 1 - Agree completely  2 - Agree a little  3 - Disagree a little  4 - Disagree completely |  |
| \|  \|  \| q54i_options \| \| --- \| --- \| --- \| | OPTIONS | \|  \| 1 \| 1 \| \| --- \| --- \| --- \| \|  \| 2 \| 2 \| \|  \| 3 \| 3 \| \|  \| 4 \| 4 \| |
| \|  \|  \| q54i (required) \| \| --- \| --- \| --- \| | 54(i). Children must be fed with a balanced diet to be healthy, and grow and develop well | \|  \| 1 \| 1 \| \| --- \| --- \| --- \| \|  \| 2 \| 2 \| \|  \| 3 \| 3 \| \|  \| 4 \| 4 \| |
| \|  \|  \| generated_note_name_301 \| \| --- \| --- \| --- \| | (Define a balanced diet as one which contains the different food groups i.e. body building foods, energy giving foods, fats, vitamins, and mineral salts) |  |
| \|  \|  \| q54 (required) \| \| --- \| --- \| --- \| | 54. Children receive **morning uji and lunch** | \|  \| 1 \| Both uji and lunch \| \| --- \| --- \| --- \| \|  \| 2 \| Lunch only \| \|  \| 3 \| Uji only \| \|  \| 4 \| No meals \| |
| \|  \|  \| q55 (required) \| \| --- \| --- \| --- \| | 55. How often do you encourage and make sure children finish all the food on their plates: *(Tick only one)* | \|  \| 1 \| Every meal \| \| --- \| --- \| --- \| \|  \| 2 \| Often I’m too busy so can only do this sometimes \| \|  \| 3 \| I never have time to do this \| \|  \| 4 \| I think it is better to let children eat on their own \| |
| \|  \|  \| q56_58_options \| \| --- \| --- \| --- \| | OPTIONS | \|  \| 1 \| Yes \| \| --- \| --- \| --- \| \|  \| 2 \| No \| |
| \|  \|  \| q56 (required) \| \| --- \| --- \| --- \| | 56. Do you plan and provide a menu with diverse foods for a day/week/month? | \|  \| 1 \| Yes \| \| --- \| --- \| --- \| \|  \| 2 \| No \| |
| \|  \|  \| q57 (required) \| \| --- \| --- \| --- \| | 57. If you don’t cook in your centre, do you advise parents on the foods to give their children to bring with them? | \|  \| 1 \| Yes \| \| --- \| --- \| --- \| \|  \| 2 \| No \| |
| \|  \|  \| q58 (required) \| \| --- \| --- \| --- \| | 58. Do you feel confident telling them which foods are appropriate? | \|  \| 1 \| Yes \| \| --- \| --- \| --- \| \|  \| 2 \| No \| |
| \|  \|  \| q59 (required) \| \| --- \| --- \| --- \| | 59. What do you **tell parents** ? |  |
| \|  \|  \| nutrition_grp_comment \| \| --- \| --- \| --- \| | **Comment** |  |
| \|  \| access_electronic_alot > wash_grp \| \| --- \| --- \| | | |
| \|  \|  \| generated_note_name_325 \| \| --- \| --- \| --- \| | ****Water, Sanitation & Hygiene (WASH)**** |  |
| \|  \|  \| generated_note_name_327 \| \| --- \| --- \| --- \| | ****Knowledge question on WASH:**** |  |
| \|  \|  \| generated_note_name_329 \| \| --- \| --- \| --- \| | 60. When do you do handwashing in the daycare center? *(tick or cross against each item)* |  |
| \|  \|  \| q60_options \| \| --- \| --- \| --- \| | OPTIONS | \|  \| 1 \| Yes \| \| --- \| --- \| --- \| \|  \| 2 \| No \| |
| \|  \|  \| q60p1 (required) \| \| --- \| --- \| --- \| | 60.1 Before preparing meals | \|  \| 1 \| Yes \| \| --- \| --- \| --- \| \|  \| 2 \| No \| |
| \|  \|  \| q60p2 (required) \| \| --- \| --- \| --- \| | 60.2 After changing diapers | \|  \| 1 \| Yes \| \| --- \| --- \| --- \| \|  \| 2 \| No \| |
| \|  \|  \| q60p3 (required) \| \| --- \| --- \| --- \| | 60.3 After handling soiled toys | \|  \| 1 \| Yes \| \| --- \| --- \| --- \| \|  \| 2 \| No \| |
| \|  \|  \| q60p4 (required) \| \| --- \| --- \| --- \| | 60.4 After visiting the toilet | \|  \| 1 \| Yes \| \| --- \| --- \| --- \| \|  \| 2 \| No \| |
| \|  \|  \| q60p5 (required) \| \| --- \| --- \| --- \| | 60.5 Other time | \|  \| 1 \| Yes \| \| --- \| --- \| --- \| \|  \| 2 \| No \| |
| \|  \| q60p5_os (required) \| \| --- \| --- \| | 60.5 Other time when hand washing is done (specify) |  |
| \|  \| q61 (required) \| \| --- \| --- \| | 61. How do you do your **handwashing** ? | \|  \| 1 \| With water only \| \| --- \| --- \| --- \| \|  \| 2 \| With water and soap \| \|  \| 3 \| No handwashing \| |
| \|  \| q62 (required) \| \| --- \| --- \| | 62. **How often** do you clean the center? | \|  \| 1 \| Once a day \| \| --- \| --- \| --- \| \|  \| 2 \| More than once a day \| \|  \| 3 \| Every other day \| |
| \|  \| q63 (required) \| \| --- \| --- \| | 63. What type of **drinking water** do you use? | \|  \| 1 \| Unboiled water \| \| --- \| --- \| --- \| \|  \| 2 \| Boiled water \| \|  \| 3 \| Water treated with water guard \| \|  \| 4 \| Bottled water \| |
| \|  \| q64 (required) \| \| --- \| --- \| | 64. How do you **dispose** of your potty/ diapers waste? | \|  \| 1 \| In the trench \| \| --- \| --- \| --- \| \|  \| 2 \| Toilet \| \|  \| 3 \| Dustbin \| \|  \| 4 \| Pit latrine \| \|  \| 5 \| Hole dug \| |
| \|  \| wash_grp_comment \| \| --- \| --- \| | **WASH Comment** |  |
| \|  \| end_time (required) \| \| --- \| --- \| | 6. End **time** |  |
| \|  \|  \|  \|  \| tl_date (required) \| \| --- \| --- \| --- \| --- \| --- \| | TL Date |  |
| \|  \|  \|  \|  \| tl_end_time (required) \| \| --- \| --- \| --- \| --- \| --- \| | END TIME |  |
| \|  \|  \|  \|  \| tl_edited (required) \| \| --- \| --- \| --- \| --- \| --- \| | Mark Complete |  |
| \|  \| generated_note_name_378 \| \| --- \| --- \| | 8.0. END OF INTERVIEW |  |
| generated_note_name_382 | YOU ARE NOT PERMITTED TO WORK ON SYSTEM TOOLS |  |
