# Supplementary material for: Developing an intervention to improve the quality of childcare centers in resource-poor urban settings: a mixed methods study in Nairobi, Kenya
Source: Front Public Health. 2023 Jul 17;11:1195460. doi: 10.3389/fpubh.2023.1195460 (PMC10387541; doi:10.3389/fpubh.2023.1195460)
Supplement: Supplementary file 2 [file Table_2.DOCX]

### ****AFRICAN POPULATION AND HEALTH RESEARCH CENTER****

#### KNOWLEDGE, MOTIVATION ATTITUDE QUESTIONNAIRE FOR CHVS

| \|  \|  \| generated_note_name_41 \| \| --- \| --- \| --- \| | ****1.0 BACKGROUND**** |  |
| --- | --- | --- | --- | --- | --- |
| \|  \|  \| cs_q1p1 (required) \| \| --- \| --- \| --- \| | 1. **Date** of interview  DON’T CHANGE THIS ENTRY |  |
| \|  \|  \| cs_q1p2 (required) \| \| --- \| --- \| --- \| | 2. **Name** of CHV: **[chv_id]** |  |
| \|  \|  \| cs_q1p3 (required) \| \| --- \| --- \| --- \| | 3. **Date of birth** of CHV |  |
| \|  \|  \| cs_q1p4 (required) \| \| --- \| --- \| --- \| | 4. **Sex** of CHV | \|  \| 1 \| Male \| \| --- \| --- \| --- \| \|  \| 2 \| Female \| |
| \|  \|  \| cs_q1p5 (required) \| \| --- \| --- \| --- \| | 5. Highest **education level** of CHV | \|  \| 1 \| None \| \| --- \| --- \| --- \| \|  \| 2 \| Primary \| \|  \| 3 \| Secondary \| \|  \| 4 \| Tertiary (including University) \| |
| \|  \| access_electronic_alot > group_ii \| \| --- \| --- \| | | |
| \|  \|  \| generated_note_name_59 \| \| --- \| --- \| --- \| | ****1.0 BACKGROUND**** |  |
| \|  \|  \| cs_q1p6 (required) \| \| --- \| --- \| --- \| | 6. **Location** | \|  \| 1 \| Korogocho \| \| --- \| --- \| --- \| \|  \| 2 \| Viwandani \| |
| \|  \|  \| interviewer (required) \| \| --- \| --- \| --- \| | 7. **Name** of interviewer | \|  \| MI18 \| Mallit Ishmael \| \| --- \| --- \| --- \| \|  \| RN18 \| Rhoda Nakhosi \| \|  \| LM18 \| Linet Mirungi \| \|  \| JO18 \| Janet Ochuodho \| \|  \| FW18 \| Florence Wairimu \| |
| \|  \|  \| cs_q1p8a (required) \| \| --- \| --- \| --- \| | 8a. Start **time** |  |
| \|  \| access_electronic_alot > knowledge_grp \| \| --- \| --- \| | | |
| \|  \|  \| access_electronic_alot > knowledge_grp > knowledge_grp_i \| \| --- \| --- \| --- \| | | |
| \|  \|  \|  \| generated_note_name_76 \| \| --- \| --- \| --- \| --- \| | ****KNOWLEDGE**** |  |
| \|  \|  \|  \| generated_note_name_79 \| \| --- \| --- \| --- \| --- \| | 1 - Agree strongly / completely  2 - Agree / Agree a little  3 - Disagree / Disagree a little  4 - Strongly disagree / Disagree completely |  |
| \|  \|  \|  \| generated_note_name_81 \| \| --- \| --- \| --- \| --- \| | ****Learning through play**** |  |
| \|  \|  \|  \| knowledge_grp_i_options \| \| --- \| --- \| --- \| --- \| | Choices | \|  \| 1 \| 1 \| \| --- \| --- \| --- \| \|  \| 2 \| 2 \| \|  \| 3 \| 3 \| \|  \| 4 \| 4 \| |
| \|  \|  \|  \| generated_note_name_85 \| \| --- \| --- \| --- \| --- \| | (Please give the options to the respondent) |  |
| \|  \|  \|  \| q9 (required) \| \| --- \| --- \| --- \| --- \| | 9 Young children learn from play | \|  \| 1 \| 1 \| \| --- \| --- \| --- \| \|  \| 2 \| 2 \| \|  \| 3 \| 3 \| \|  \| 4 \| 4 \| |
| \|  \|  \|  \| q10 (required) \| \| --- \| --- \| --- \| --- \| | 10 Centers need to provide space, toys and time for children to play | \|  \| 1 \| 1 \| \| --- \| --- \| --- \| \|  \| 2 \| 2 \| \|  \| 3 \| 3 \| \|  \| 4 \| 4 \| |
| \|  \|  \|  \| generated_note_name_92 \| \| --- \| --- \| --- \| --- \| | ****Child protection/Child safety/Abuse/positive discipline/responsive caregiving**** |  |
| \|  \|  \|  \| q11 (required) \| \| --- \| --- \| --- \| --- \| | 11. Children must be **handled harshly** for them to develop better  (Harshly means shouting, physical beating, yelling, or any form of force used to handle children) | \|  \| 1 \| Always \| \| --- \| --- \| --- \| \|  \| 2 \| Sometimes \| \|  \| 3 \| Never \| |
| \|  \|  \| access_electronic_alot > knowledge_grp > knowledge_grp_ii \| \| --- \| --- \| --- \| | | |
| \|  \|  \|  \| q12 (required) \| \| --- \| --- \| --- \| --- \| | 12. How should children who misbehave be **corrected** ?  (Tick all that apply) | \|  \| 1 \| Using Physical punishment \| \| --- \| --- \| --- \| \|  \| 2 \| Using Verbal punishment \| \|  \| 3 \| Distract child with another activity \| \|  \| 4 \| Explain wrong deeds to child calmly \| |
| \|  \|  \|  \| generated_note_name_103 \| \| --- \| --- \| --- \| --- \| | ****13. The following communication/ interaction practices with children are appropriate**** |  |
| \|  \|  \|  \| q4_options \| \| --- \| --- \| --- \| --- \| | Choices | \|  \| 1 \| Yes \| \| --- \| --- \| --- \| \|  \| 2 \| No \| |
| \|  \|  \|  \| q13p1 (required) \| \| --- \| --- \| --- \| --- \| | 13.1 Very little talk or no conversation needed | \|  \| 1 \| Yes \| \| --- \| --- \| --- \| \|  \| 2 \| No \| |
| \|  \|  \|  \| q13p2 (required) \| \| --- \| --- \| --- \| --- \| | 13.2 Shouting at/ speaking angrily at the children/ with irritation | \|  \| 1 \| Yes \| \| --- \| --- \| --- \| \|  \| 2 \| No \| |
| \|  \|  \|  \| q13p3 (required) \| \| --- \| --- \| --- \| --- \| | 13.3 Showing no interest in children’s conversation | \|  \| 1 \| Yes \| \| --- \| --- \| --- \| \|  \| 2 \| No \| |
| \|  \|  \|  \| q13p4 (required) \| \| --- \| --- \| --- \| --- \| | 13.4 Allowing the child to express themselves freely in the conversation | \|  \| 1 \| Yes \| \| --- \| --- \| --- \| \|  \| 2 \| No \| |
| \|  \|  \| q14 (required) \| \| --- \| --- \| --- \| | 14. **Children’s needs** (E.g. if a child cries or sleepy or hungry) must be responded to  (Please give the options to the respondent) | \|  \| 1 \| Immediately \| \| --- \| --- \| --- \| \|  \| 2 \| Anytime \| \|  \| 3 \| Never \| |
| \|  \|  \| access_electronic_alot > knowledge_grp > nutrition_grp \| \| --- \| --- \| --- \| | | |
| \|  \|  \|  \| generated_note_name_124 \| \| --- \| --- \| --- \| --- \| | ****Nutrition**** |  |
| \|  \|  \|  \| generated_note_name_126 \| \| --- \| --- \| --- \| --- \| | (Please give the options to the respondent) |  |
| \|  \|  \|  \| q15 (required) \| \| --- \| --- \| --- \| --- \| | 15 Children should be **fed** with: | \|  \| 1 \| Same foods everyday \| \| --- \| --- \| --- \| \|  \| 2 \| Variety of foods rotated on different days \| |
| \|  \|  \|  \| generated_note_name_130 \| \| --- \| --- \| --- \| --- \| | 1 - Agree completely  2 - Agree a little  3 - Disagree a little  4 - Disagree completely |  |
| \|  \|  \|  \| nutrition_grp_label \| \| --- \| --- \| --- \| --- \| | Options | \|  \| 1 \| 1 \| \| --- \| --- \| --- \| \|  \| 2 \| 2 \| \|  \| 3 \| 3 \| \|  \| 4 \| 4 \| |
| \|  \|  \|  \| q16 (required) \| \| --- \| --- \| --- \| --- \| | 16 It is important that children are served **warm (not cold) food** | \|  \| 1 \| 1 \| \| --- \| --- \| --- \| \|  \| 2 \| 2 \| \|  \| 3 \| 3 \| \|  \| 4 \| 4 \| |
| \|  \|  \|  \| q17 (required) \| \| --- \| --- \| --- \| --- \| | 17 Caregivers should have **knowledge** about a **balanced diet** , and the different food groups | \|  \| 1 \| 1 \| \| --- \| --- \| --- \| \|  \| 2 \| 2 \| \|  \| 3 \| 3 \| \|  \| 4 \| 4 \| |
| \|  \|  \| access_electronic_alot > knowledge_grp > health_grp \| \| --- \| --- \| --- \| | | |
| \|  \|  \|  \| generated_note_name_143 \| \| --- \| --- \| --- \| --- \| | ****Health**** |  |
| \|  \|  \|  \| generated_note_name_145 \| \| --- \| --- \| --- \| --- \| | 1 - Agree completely  2 - Agree a little  3 - Disagree a little  4 - Disagree completely |  |
| \|  \|  \|  \| q18_grp_options \| \| --- \| --- \| --- \| --- \| | Options | \|  \| 1 \| Correct \| \| --- \| --- \| --- \| \|  \| 2 \| Wrong \| |
| \|  \|  \|  \| q18 (required) \| \| --- \| --- \| --- \| --- \| | 18 It is important to know about the **immunization status** of children in day cares | \|  \| 1 \| Agree completely \| \| --- \| --- \| --- \| \|  \| 2 \| Agree a little \| \|  \| 3 \| Disagree a little \| \|  \| 4 \| Disagree completely \| |
| \|  \|  \|  \| generated_note_name_151 \| \| --- \| --- \| --- \| --- \| | 19 (i) Can you tell me which **vaccinations** children should have and when? |  |
| \|  \|  \|  \| q19_grp_options \| \| --- \| --- \| --- \| --- \| | Options | \|  \| 1 \| Correct \| \| --- \| --- \| --- \| \|  \| 2 \| Wrong \| |
| \|  \|  \|  \| q19p1 (required) \| \| --- \| --- \| --- \| --- \| | 19.1 Birth (BCG & Polio 0) | \|  \| 1 \| Correct \| \| --- \| --- \| --- \| \|  \| 2 \| Wrong \| |
| \|  \|  \|  \| q19p2 (required) \| \| --- \| --- \| --- \| --- \| | 19.2 6 weeks (DPT1 and Polio 1) | \|  \| 1 \| Correct \| \| --- \| --- \| --- \| \|  \| 2 \| Wrong \| |
| \|  \|  \|  \| q19p3 (required) \| \| --- \| --- \| --- \| --- \| | 19.3 10 weeks(DPT2 and Polio 2) | \|  \| 1 \| Correct \| \| --- \| --- \| --- \| \|  \| 2 \| Wrong \| |
| \|  \|  \|  \| q19p4 (required) \| \| --- \| --- \| --- \| --- \| | 19.4 14 weeks (DPT3 and Polio 3) | \|  \| 1 \| Correct \| \| --- \| --- \| --- \| \|  \| 2 \| Wrong \| |
| \|  \|  \|  \| q19p5 (required) \| \| --- \| --- \| --- \| --- \| | 19.5 9 months (measles) | \|  \| 1 \| Correct \| \| --- \| --- \| --- \| \|  \| 2 \| Wrong \| |
| \|  \|  \| access_electronic_alot > knowledge_grp > health_grp_ii \| \| --- \| --- \| --- \| | | |
| \|  \|  \|  \| generated_note_name_170 \| \| --- \| --- \| --- \| --- \| | ****Health cont.**** |  |
| \|  \|  \|  \| generated_note_name_172 \| \| --- \| --- \| --- \| --- \| | 1 - Agree strongly  2 - Agree  3 - Disagree  4 - Strongly disagree |  |
| \|  \|  \|  \| health_grp_ii_options \| \| --- \| --- \| --- \| --- \| | Options | \|  \| 1 \| 1 \| \| --- \| --- \| --- \| \|  \| 2 \| 2 \| \|  \| 3 \| 3 \| \|  \| 4 \| 4 \| |
| \|  \|  \|  \| q20 (required) \| \| --- \| --- \| --- \| --- \| | 20 There should be daily health check and an understanding of what to do if a child is sick | \|  \| 1 \| 1 \| \| --- \| --- \| --- \| \|  \| 2 \| 2 \| \|  \| 3 \| 3 \| \|  \| 4 \| 4 \| |
| \|  \|  \|  \| q21 (required) \| \| --- \| --- \| --- \| --- \| | 21 Center providers should be knowledgeable on how to conduct first aid in case of an emergency | \|  \| 1 \| 1 \| \| --- \| --- \| --- \| \|  \| 2 \| 2 \| \|  \| 3 \| 3 \| \|  \| 4 \| 4 \| |
| \|  \|  \|  \| generated_note_name_181 \| \| --- \| --- \| --- \| --- \| | 22 Mention **three examples of emergencies** , that may require First aid in a day care center |  |
| \|  \|  \|  \| q22p1 (required) \| \| --- \| --- \| --- \| --- \| | Emergency 1 |  |
| \|  \|  \|  \| q22p2 (required) \| \| --- \| --- \| --- \| --- \| | Emergency 2 |  |
| \|  \|  \|  \| q22p3 (required) \| \| --- \| --- \| --- \| --- \| | Emergency 3 |  |
| \|  \|  \| access_electronic_alot > knowledge_grp > wash_grp \| \| --- \| --- \| --- \| | | |
| \|  \|  \|  \| generated_note_name_194 \| \| --- \| --- \| --- \| --- \| | ****WASH**** |  |
| \|  \|  \|  \| generated_note_name_197 \| \| --- \| --- \| --- \| --- \| | ****Knowledge questions on WASH**** |  |
| \|  \|  \|  \| q23 (required) \| \| --- \| --- \| --- \| --- \| | 23 When should **handwashing** be done? Handwashing should be done (tick or cross against each item) | \|  \| 1 \| Before preparing meals \| \| --- \| --- \| --- \| \|  \| 2 \| After changing diapers \| \|  \| 3 \| After handling soiled toys \| |
| \|  \|  \|  \| q24 (required) \| \| --- \| --- \| --- \| --- \| | 24 How do you do **your handwashing** ? | \|  \| 1 \| With water only \| \| --- \| --- \| --- \| \|  \| 2 \| With water and soap \| \|  \| 3 \| No handwashing \| |
| \|  \|  \|  \| generated_note_name_203 \| \| --- \| --- \| --- \| --- \| | 1 - Agree strongly / completely  2 - Agree / Agree a little  3 - Disagree / Disagree a little  4 - Strongly disagree / Disagree completely |  |
| \|  \|  \|  \| wash_grp_options \| \| --- \| --- \| --- \| --- \| | OPTIONS | \|  \| 1 \| 1 \| \| --- \| --- \| --- \| \|  \| 2 \| 2 \| \|  \| 3 \| 3 \| \|  \| 4 \| 4 \| |
| \|  \|  \|  \| q25 (required) \| \| --- \| --- \| --- \| --- \| | 25. Young children need to have their hands washed with soap **before feeding** | \|  \| 1 \| 1 \| \| --- \| --- \| --- \| \|  \| 2 \| 2 \| \|  \| 3 \| 3 \| \|  \| 4 \| 4 \| |
| \|  \|  \|  \| q26 (required) \| \| --- \| --- \| --- \| --- \| | 26 It is ok for young children in a day care center to **share feeding utensils** while they are feeding | \|  \| 1 \| Yes \| \| --- \| --- \| --- \| \|  \| 2 \| No \| |
| \|  \| access_electronic_alot > practice_grp \| \| --- \| --- \| | | |
| \|  \|  \| access_electronic_alot > practice_grp > support_and_supervision_grp \| \| --- \| --- \| --- \| | | |
| \|  \|  \|  \| generated_note_name_219 \| \| --- \| --- \| --- \| --- \| | ****PRACTICE IN SUPPORTING CHILD CARE**** |  |
| \|  \|  \|  \| generated_note_name_222 \| \| --- \| --- \| --- \| --- \| | ****Providing support supervision and mentoring of center providers**** |  |
| \|  \|  \|  \| q27 (required) \| \| --- \| --- \| --- \| --- \| | 27 **How often** do you visit child care centers | \|  \| 1 \| Weekly \| \| --- \| --- \| --- \| \|  \| 2 \| 1-3 times a monthly \| \|  \| 3 \| Less than once a month \| \|  \| 4 \| Never \| |
| \|  \|  \| access_electronic_alot > practice_grp > support_and_supervision_grp_ii \| \| --- \| --- \| --- \| | | |
| \|  \|  \|  \| q28 (required) \| \| --- \| --- \| --- \| --- \| | 28 **How much time** do you spend at the child care centers when you visit | \|  \| 1 \| Up to 30 mins \| \| --- \| --- \| --- \| \|  \| 2 \| 30 - 60 mins \| \|  \| 3 \| More than an hour \| |
| \|  \|  \|  \| q29 (required) \| \| --- \| --- \| --- \| --- \| | 29 **Do you support/advise** center providers ? | \|  \| 1 \| Yes \| \| --- \| --- \| --- \| \|  \| 2 \| No \| |
| \|  \|  \| access_electronic_alot > practice_grp > q6p14_grp \| \| --- \| --- \| --- \| | | |
| \|  \|  \|  \| q29b (required) \| \| --- \| --- \| --- \| --- \| | 29b Please **list the key messages you convey** , or the main things you check and provide advice on : |  |
| \|  \|  \|  \| q29c (required) \| \| --- \| --- \| --- \| --- \| | 29c **How often** do you support them in? | \|  \| 1 \| Weekly \| \| --- \| --- \| --- \| \|  \| 2 \| Monthly \| \|  \| 3 \| Less than once a month \| |
| \|  \|  \| access_electronic_alot > practice_grp > attitude_and_motivation \| \| --- \| --- \| --- \| | | |
| \|  \|  \|  \| generated_note_name_253 \| \| --- \| --- \| --- \| --- \| | ****ATTITUDE/ MOTIVATION AND PERCIEVED COMPETENCE TO SUPPORT SUPERVISE CENTRE PROVIDERS**** |  |
| \|  \|  \|  \| q30 (required) \| \| --- \| --- \| --- \| --- \| | 30. Do you feel **motivated to support day-care centers** within your role as a CHV? | \|  \| 1 \| Strongly motivated \| \| --- \| --- \| --- \| \|  \| 2 \| Motivated \| \|  \| 3 \| Somehow motivated \| \|  \| 4 \| Not motivated \| |
| \|  \|  \|  \| q31 (required) \| \| --- \| --- \| --- \| --- \| | 31 **Do you feel confident** to support day-care providers to improve the quality of their day-care? | \|  \| 1 \| Yes \| \| --- \| --- \| --- \| \|  \| 2 \| No \| \|  \| 98 \| Not sure \| |
| \|  \|  \|  \| q32 (required) \| \| --- \| --- \| --- \| --- \| | 32 Do you feel you have the **required competencies** to support day-care providers to improve the health and development of children in their care? | \|  \| 1 \| I am very competent \| \| --- \| --- \| --- \| \|  \| 2 \| I have some of the competencies required \| \|  \| 3 \| I don’t feel I have the required competencies \| \|  \| 98 \| I don’t know \| |
| \|  \|  \| access_electronic_alot > practice_grp > opportunities_and_barriers \| \| --- \| --- \| --- \| | | |
| \|  \|  \|  \| generated_note_name_268 \| \| --- \| --- \| --- \| --- \| | ****OPPORTUNITY / BARRIERS**** |  |
| \|  \|  \|  \| q33 (required) \| \| --- \| --- \| --- \| --- \| | 33 What are the **barriers** you face in supporting child care centers?  Tick all that apply | \|  \| 1 \| Lack of time due to workload \| \| --- \| --- \| --- \| \|  \| 2 \| Lack of training \| \|  \| 3 \| Lack of motivation \| \|  \| 4 \| Little interest of day-care providers \| \|  \| 5 \| Lack of tools \| \|  \| 6 \| Lack of guidance \| \|  \| 96 \| Other (specify) \| |
| \|  \|  \| q33_os (required) \| \| --- \| --- \| --- \| | 33 Other **(specify)** |  |
| \|  \| cs_q1p8b (required) \| \| --- \| --- \| | 8b. End **time** |  |
| \|  \|  \|  \|  \| tl_end_time (required) \| \| --- \| --- \| --- \| --- \| --- \| | END TIME |  |
| \|  \|  \|  \|  \| tl_edited (required) \| \| --- \| --- \| --- \| --- \| --- \| | Mark Complete |  |
| \|  \| generated_note_name_301 \| \| --- \| --- \| | 8.0. END OF INTERVIEW |  |
| generated_note_name_305 | YOU ARE NOT PERMITTED TO WORK ON SYSTEM TOOLS |  |
