# Supplementary material for: Developing an intervention to improve the quality of childcare centers in resource-poor urban settings: a mixed methods study in Nairobi, Kenya
Source: Front Public Health. 2023 Jul 17;11:1195460. doi: 10.3389/fpubh.2023.1195460 (PMC10387541; doi:10.3389/fpubh.2023.1195460)
Supplement: Supplementary file 3 [file Table_3.DOCX]

**African Population and Health Research Center**

Community of Practice project

**TOOL 4: CoP supportive supervision assessment tool**

| \|  \|  \| generated_note_name_39 \| \| --- \| --- \| --- \| | ****Section1: Centre profile**** |  |
| --- | --- | --- | --- | --- | --- |
| \|  \|  \| interviewer (required) \| \| --- \| --- \| --- \| | ii. **Name** of interviewer |  |
| \|  \|  \| start_time (required) \| \| --- \| --- \| --- \| | iii. Start **time** |  |
| \|  \|  \| q1 (required) \| \| --- \| --- \| --- \| | 1. Day care centre: |  |
| \|  \|  \| q2 (required) \| \| --- \| --- \| --- \| | 2. Center provider ID |  |
| \|  \|  \| q3 (required) \| \| --- \| --- \| --- \| | 3. CHV |  |
| \|  \|  \| q4 (required) \| \| --- \| --- \| --- \| | 4. **Location** of centre | \|  \| 1 \| Korogocho \| \| --- \| --- \| --- \| \|  \| 2 \| Viwandani \| |
| \|  \|  \| q5 (required) \| \| --- \| --- \| --- \| | 5. **Date** of interview/observation  DON’T CHANGE THIS ENTRY |  |
| \|  \| access_electronic_alot > q6_q8_grp \| \| --- \| --- \| | | |
| \|  \|  \| generated_note_name_62 \| \| --- \| --- \| --- \| | ****Learning through play****  (3 quality points) |  |
| \|  \|  \| generated_note_name_64 \| \| --- \| --- \| --- \| | *Observe items or ask the provider as necessary* |  |
| \|  \|  \| q6_options \| \| --- \| --- \| --- \| | OPTIONS | \|  \| 1 \| Yes \| \| --- \| --- \| --- \| \|  \| 2 \| No \| |
| \|  \|  \| q6 (required) \| \| --- \| --- \| --- \| | 6. Presence of a **well labelled learning centre** | \|  \| 1 \| Yes \| \| --- \| --- \| --- \| \|  \| 2 \| No \| |
| \|  \|  \| q7 (required) \| \| --- \| --- \| --- \| | 7. **Daily schedule** of play times posted and utilised | \|  \| 1 \| Yes \| \| --- \| --- \| --- \| \|  \| 2 \| No \| |
| \|  \|  \| q8 (required) \| \| --- \| --- \| --- \| | 8. Each child has **something to play with** | \|  \| 1 \| Yes \| \| --- \| --- \| --- \| \|  \| 2 \| No \| |
| \|  \| access_electronic_alot > q9_q17_grp \| \| --- \| --- \| | | |
| \|  \|  \| generated_note_name_83 \| \| --- \| --- \| --- \| | ****Health, WASH & Nutrition****  (9 quality points) |  |
| \|  \|  \| generated_note_name_85 \| \| --- \| --- \| --- \| | *Observe items or ask the provider as necessary* |  |
| \|  \|  \| q9_options \| \| --- \| --- \| --- \| | OPTIONS | \|  \| 1 \| Yes \| \| --- \| --- \| --- \| \|  \| 2 \| No \| |
| \|  \|  \| q9 (required) \| \| --- \| --- \| --- \| | 9. Daily health check and knowledge of what to do if a child is unwell | \|  \| 1 \| Yes \| \| --- \| --- \| --- \| \|  \| 2 \| No \| |
| \|  \|  \| q10 (required) \| \| --- \| --- \| --- \| | 10. First aid kit & knowledge on use | \|  \| 1 \| Yes \| \| --- \| --- \| --- \| \|  \| 2 \| No \| |
| \|  \|  \| q11 (required) \| \| --- \| --- \| --- \| | 11. Visibly displayed routine immunisation schedule | \|  \| 1 \| Yes \| \| --- \| --- \| --- \| \|  \| 2 \| No \| |
| \|  \|  \| q12 (required) \| \| --- \| --- \| --- \| | 12. Availability of handwashing facility with soap in use | \|  \| 1 \| Yes \| \| --- \| --- \| --- \| \|  \| 2 \| No \| |
| \|  \|  \| q13 (required) \| \| --- \| --- \| --- \| | 13. Availability of clean & sufficient potties(one potty per 5 children) | \|  \| 1 \| Yes \| \| --- \| --- \| --- \| \|  \| 2 \| No \| |
| \|  \|  \| q14 (required) \| \| --- \| --- \| --- \| | 14. Centre is visibly clean | \|  \| 1 \| Yes \| \| --- \| --- \| --- \| \|  \| 2 \| No \| |
| \|  \|  \| q15 (required) \| \| --- \| --- \| --- \| | 15. Access to clean drinking water | \|  \| 1 \| Yes \| \| --- \| --- \| --- \| \|  \| 2 \| No \| |
| \|  \|  \| q16 (required) \| \| --- \| --- \| --- \| | 16. Children have breakfast and lunch | \|  \| 1 \| Yes \| \| --- \| --- \| --- \| \|  \| 2 \| No \| |
| \|  \|  \| q17 (required) \| \| --- \| --- \| --- \| | 17. Food menu posted with options | \|  \| 1 \| Yes \| \| --- \| --- \| --- \| \|  \| 2 \| No \| |
| \|  \| access_electronic_alot > q18_q22_grp \| \| --- \| --- \| | | |
| \|  \|  \| generated_note_name_113 \| \| --- \| --- \| --- \| | ****Child Protection, Abuse, Positive Discipline and Child Safety****  (5 quality points) |  |
| \|  \|  \| generated_note_name_115 \| \| --- \| --- \| --- \| | *Observe items or ask the provider as necessary* |  |
| \|  \|  \| q18_options \| \| --- \| --- \| --- \| | OPTIONS | \|  \| 1 \| Yes \| \| --- \| --- \| --- \| \|  \| 2 \| No \| |
| \|  \|  \| q18 (required) \| \| --- \| --- \| --- \| | 18. Understand child protection issues | \|  \| 1 \| Yes \| \| --- \| --- \| --- \| \|  \| 2 \| No \| |
| \|  \|  \| q19 (required) \| \| --- \| --- \| --- \| | 19. Able to recognize all forms child abuse | \|  \| 1 \| Yes \| \| --- \| --- \| --- \| \|  \| 2 \| No \| |
| \|  \|  \| q20 (required) \| \| --- \| --- \| --- \| | 20. Understand ways to discipline children without using force | \|  \| 1 \| Yes \| \| --- \| --- \| --- \| \|  \| 2 \| No \| |
| \|  \|  \| q21 (required) \| \| --- \| --- \| --- \| | 21. Understand alternatives to corporal punishment | \|  \| 1 \| Yes \| \| --- \| --- \| --- \| \|  \| 2 \| No \| |
| \|  \|  \| q22 (required) \| \| --- \| --- \| --- \| | 22. Able to recognise the effects of child abuse | \|  \| 1 \| Yes \| \| --- \| --- \| --- \| \|  \| 2 \| No \| |
| \|  \| access_electronic_alot > q23_q24_grp \| \| --- \| --- \| | | |
| \|  \|  \| generated_note_name_136 \| \| --- \| --- \| --- \| | ****Business and Administration****  (2 quality points) |  |
| \|  \|  \| generated_note_name_138 \| \| --- \| --- \| --- \| | *Observe items or ask the provider as necessary* |  |
| \|  \|  \| q23_options \| \| --- \| --- \| --- \| | OPTIONS | \|  \| 1 \| Yes \| \| --- \| --- \| --- \| \|  \| 2 \| No \| |
| \|  \|  \| q23 (required) \| \| --- \| --- \| --- \| | 23. Track attendance daily | \|  \| 1 \| Yes \| \| --- \| --- \| --- \| \|  \| 2 \| No \| |
| \|  \|  \| q24 (required) \| \| --- \| --- \| --- \| | 24. Track finances daily/ weekly / monthly | \|  \| 1 \| Yes \| \| --- \| --- \| --- \| \|  \| 2 \| No \| |
| \|  \| end_time (required) \| \| --- \| --- \| | 6. End **time** |  |
| \|  \| generated_note_name_178 \| \| --- \| --- \| | **Total score is :** [total_score] |  |
| \|  \| js_hh_tl_consent (required) \| \| --- \| --- \| | Are you the TL?  Choose "NO" if you are not the Team Leader | \|  \| 1 \| Yes \| \| --- \| --- \| --- \| \|  \| 2 \| No \| |
| \|  \| access_electronic_alot > js_tl_grp \| \| --- \| --- \| | | |
| \|  \|  \|  \|  \| tl_date (required) \| \| --- \| --- \| --- \| --- \| --- \| | TL Date |  |
| \|  \|  \|  \|  \| tl_end_time (required) \| \| --- \| --- \| --- \| --- \| --- \| | END TIME |  |
| \|  \|  \|  \|  \| tl_edited (required) \| \| --- \| --- \| --- \| --- \| --- \| | Mark Complete |  |
| \|  \| generated_note_name_195 \| \| --- \| --- \| | 8.0. END OF INTERVIEW |  |
| generated_note_name_199 | YOU ARE NOT PERMITTED TO WORK ON SYSTEM TOOLS |  |
