# Supplementary material for: Developing an intervention to improve the quality of childcare centers in resource-poor urban settings: a mixed methods study in Nairobi, Kenya
Source: Front Public Health. 2023 Jul 17;11:1195460. doi: 10.3389/fpubh.2023.1195460 (PMC10387541; doi:10.3389/fpubh.2023.1195460)
Supplement: Supplementary file 5 [file Data_Sheet_1.docx]

**Supplementary material Table 8: Stakeholder perspectives on topics for inclusion in the training and CoP sessions**

| **Priority TOPICS chosen after discussions with Kidogo team** | **Possible areas to focus training for CHV/centre providers** | **Areas of ECD addressed in the past by sub-county/CHVs/ other projects** | **Areas of training specified by CHVs & sub/ county during CoP workshops held in August** | **Areas of training requested by centre providers** | **Areas of training requested by parents** |
| --- | --- | --- | --- | --- | --- |
| Safe and stimulating environment | Child protection, safety and security  Emotional Environment: the emotions of the children who spend time in day-care centres, the staff that work there and the parents who leave their children there  Outdoor Environment: access to outdoor space on a daily basis – regardless of all except the worst weather. It allows children to use all of their senses to appreciate the colours, different noises, the sense of space and of scale  Indoor Environment: It’s comfortable, safe, interesting, attractive and appropriate for the child or children who use it | Trainings were provided on play and stimulation with care givers, centre managers and selected CHVs by Concern and the sub-county. | Summary of key areas for training  -Early identification of disabilities such as hearing impairment and autism  -Growth monitoring and importance of immunization  -Sanitation  -Early referral  -Training on emerging and re-emerging diseases  -Child rights and protection  -Infection prevention/control e.g. Covid-19  -Train on income generating activities. -Self-referral of the children under 5 years  -Care for and management of children in case of emergencies  -Train on how and when to change children’s clothes or diapers  -Train on skills to give the sick children medication (parents leave sick children with medicine and caregivers may not know how to administer the medication & the dosage). | -How to handle children in different situations  -Good nutrition/feeding  -How to identify children in danger  -Maintaining cleanliness and hygiene  - feeding program so that I can be able to consistently serve the children with the right meals, the balanced diet.  -How to keep children safe  -how to know which materials to use and also training on the new curriculum with current ECD so we need to have skills on that | -Providers to stop threatening their children  -Feeding children well  -Child health  -Learn how to talk and respect parents  - playing items, a spacious house where the older and little children can play well  -First Aid or providing first aid  -How to store water safely or get regular supply of water enough for the children so they don’t go thirsty  -Hygiene  -How to talk to parents  -How to be friendly with the children and caring for them  -Investing in play materials  -Maintaining cleanliness  - Some parents think children should be beaten “if necessary” so education to the provider, and for the provider to tell parents on other ways to teach behaviour.  ECD - playing materials, some don’t have any or don’t want to buy, so making their own out of recycled materials would work.  Provider should know how to treat all children equally and ensure their needs come first. |
| Health, nutrition and WASH | Immunizations schedule for children  Prevention of other communicable diseases  How to identify danger signs among the children and how to manage it  Ensuring good nutrition with a comprehensive menu that provides variety of choices  Essential nutrients including proteins, vitamins, calcium etc. included in meals for children  Access to clean drinking water  Handwashing facility with soap available and in use  Centre is cleaned daily & has sufficient potties for the number of children accommodated | Childcare centres have been given nutrition support through “Malezi Bora” which is nutrition program of the Sub-county.  Training was given to the centre care providers on nurturing care and nutrition.  The Korogocho project that was supported by Concern Worldwide has been doing nutrition, WASH, Vitamin A supplementation, deworming and MUAC screening for under five  Aqua tabs to purify drinking water in collaboration with an organization called Afya wa jijini  Health education on the importance of ventilation e.g. there was a case of a childcare centre in Maringo that did not have windows. | Sanitation is not good in some child care centres so there should be training because sometimes the CHVs encounter childcares that have very dire hygiene conditions so additional training will be very useful  Training mothers and centre managers on early detection of malnutrition in under five using the MUAC tape will be very important  The tools used are Ministry of Health (MoH) tools and guidelines which are the general guidelines used for children under five. There are specific indicators for specific levels. For example, the childcare is marked under a comprehensive tool for the community but there is no specific indicator for “childcare centres.”  The 514-reporting tool has some major indicators that the CHVs use to report. However, there are no indicators on stimulation, developmental milestones, disability and play. The CHVs have been trained on these areas and if they are added to the 514, and the mother-child booklet they can capture these alongside their work.  The best idea would be to add to the existing tool and not introduce a new one and the additions should also not be too many (for ease of reporting). It will be useful to review the existing indicators and see which are missing and add them. | -Potty training | Feeding the children is a real challenge for many parents, some providers are providing food if parents don’t send their children with enough - so education to the provider on how to use meagre resources to provide the most nutritious meal they can to needy children. Feeding at the right time mentioned too.  Some providers are said to need training on hygiene i.e. regular diaper changing, hand washing. Parents check if there is water and soap available.  One parent recommended a small pharmacy in the day-care to treat minor illnesses, fever, teething |
| Management Administration | Daily checks of children attendance and payment issues  Looking at expenditures and keeping basic accounts |  | Parents sometimes don’t pay  Financial management is a key component of day-care business and some CHVs are also childcare centre owners so they can benefit from the training and improve their finances  The care providers lack technical capacity on basic skills | -How to improve my childcare to fit the modern way of running childcare in order to attract more clients to come so that it will increase the number of children coming.  - Also how I can be able to sustain the care givers so they can continue giving quality service to the children here |  |
